# Spatial and Seasonal Structuring of Bacterial Communities and Putative Pathogen‐Associated Bacterial Taxa at Four Korean Coastal Locations

**DOI:** 10.1111/1758-2229.70431

**Published:** 2026-11-03

**Authors:** Yu Jin Kim, Min‐Jeong Kim, Seon Min Kim, Hyun Ju Ha, Joon Sang Park, I. K. A. Agus Rini, Sukchan Lee, Taek‐Kyun Lee

**Affiliations:** ^1^ Ecological Risk Research Department Korea Institute of Ocean Science & Technology Geoje Republic of Korea; ^2^ Department of Ocean Science University of Science and Technology Daejeon Republic of Korea; ^3^ Library of Marine Samples, Korea Institute of Ocean Science and Technology Geoje Republic of Korea; ^4^ Department of Integrative Biotechnology Sungkyunkwan University Suwon Republic of Korea

**Keywords:** 16S rRNA gene sequencing, bacterial communities, Korean coastal waters, microbial baseline, putative pathogen‐associated taxa, spatial differentiation

## Abstract

Coastal marine environments are dynamic transition zones where offshore water masses and terrestrial inputs shape microbial community structure. However, standardized comparisons of bacterial communities across geographically distinct Korean coastal waters remain limited, constraining the development of microbial baselines for long‐term monitoring. Surface seawater bacterial communities from four Korean coastal locations were analysed using 16S rRNA gene amplicon sequencing. Total bacterial communities and putative pathogen‐associated bacterial taxa (PPB) assemblages differed among the four sampled locations and between the March and September sampling periods. PERMANOVA revealed significant effects of sampling location, sampling period and their interaction on community composition. Although Pseudomonadota dominated across all sampled locations, class‐ and genus‐level community compositions differed markedly among sites. *Vibrio* was a major component of PPB assemblages, while SIMPER analysis indicated that compositional differences among the sampled locations were associated with variation in combinations of co‐occurring taxa. Putative species‐level assignments further suggested location‐associated distribution patterns among dominant PPB taxa. Despite the absence of concurrent environmental metadata, these findings provide preliminary location‐specific baseline data for future coastal microbial monitoring and studies of environmental change.

## Graphical abstract

Surface seawater bacterial communities across four Korean coastal locations exhibited differences in community composition among sampling locations and between the March and September sampling periods. Putative pathogen‐associated bacterial taxa (PPB), particularly *Vibrio* and co‐occurring taxa, showed location‐associated variation in relative abundance, providing preliminary baseline information for future coastal monitoring.

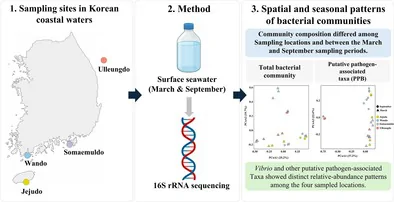

## 1 Introduction

The ocean covers approximately 70% of the Earth's surface and represents the largest ecosystem on the planet. Microorganisms are key components sustaining biogeochemical cycling and energy flow in marine environments. In particular, marine bacteria play central roles in the degradation of organic matter and the cycling of major elements such as carbon, nitrogen and phosphorus, thereby contributing directly to marine productivity and ecosystem functioning (Falkowski et al. 2008; Moran 2015). Coastal waters are dynamic transition zones where offshore water masses interact with freshwater inflow and terrestrially derived organic matter (Paerl et al. 2011; Cloern et al. 2016). These environmental gradients strongly influence bacterial community structure and coastal bacterial assemblages are known to respond sensitively to physicochemical variability. In addition, geographic characteristics, hydrodynamic connectivity, mixing intensity and local coastal environmental conditions have been associated with spatial variation in bacterial community composition across marine ecosystems (Fuhrman et al. 2015; Sunagawa et al. 2015). Understanding microbial community variation among coastal ecosystems is important for establishing ecological baselines and supporting long‐term environmental monitoring efforts.

In coastal environments, several bacterial taxa, including members of the genus *Vibrio*, have been associated with opportunistic pathogenicity, and their distributions have been reported to be associated with environmental variability in previous studies, elevated organic matter concentrations and coastal environmental change (Vezzulli et al. 2016; Baker‐Austin et al. 2017). In particular, non‐cholera *Vibrio* species have been recognized as climate‐sensitive taxa responsive to environmental fluctuations in coastal systems (Trinanes and Martinez‐Urtaza 2021). However, because 16S rRNA gene‐based analyses cannot reliably distinguish pathogenic from non‐pathogenic strains within the same genus, recent studies have increasingly focused on the ecological distribution of putative pathogen‐associated bacterial taxa (PPB) rather than direct detection of specific pathogens (Han et al. 2021; Yu and Rhee 2026).

Recent advances in 16S rRNA gene metabarcoding have enabled standardized comparisons of bacterial community structure across geographically distinct regions. These approaches allow not only diversity assessments but also quantitative evaluation of taxa contributing to community dissimilarity through SIMPER analysis (Clarke 1993) and identification of region‐specific taxa using indicator species analysis (Dufrêne and Legendre 1997). Although numerous microbial studies have been conducted in Korean coastal waters (Suh et al. 2015; Kim et al. 2023), most have focused on individual stations or geographically restricted regions. Standardized regional comparisons of bacterial communities across geographically distinct Korean coastal waters remain relatively limited. Accordingly, the present study aimed to establish preliminary, location‐specific baseline data describing bacterial community composition at the four sampled Korean coastal locations, thereby providing a foundation for future coastal microbial monitoring, environmental change assessments and studies integrating concurrent environmental measurements.

Therefore, this study investigated four Korean coastal regions representing contrasting geographic settings: Jejudo (Seogwipo; offshore‐influenced waters), Wando (coastal and aquaculture‐influenced waters), Somaemuldo (offshore–coastal transitional waters) and Ulleungdo (oceanic and geographically isolated waters). Surface seawater bacterial communities were analysed during March and September. The objectives of this study were to: (1) compare α‐ and β‐diversity patterns of total bacterial communities and PPB assemblages; (2) evaluate regional and seasonal variation in community composition among regions and (3) identify taxa associated with regional community differentiation using SIMPER and indicator species analyses. Rather than resolving specific environmental drivers of community variation, the present study aimed to provide preliminary location‐specific baseline data describing bacterial community composition at the four sampled Korean coastal locations. Such location‐specific baseline data may provide a foundation for future coastal microbial monitoring, ecosystem assessment and investigations of environmental change (Sunagawa et al. 2015; Logares et al. 2020).

## 2 Materials and Methods

### 2.1 Study Area and Sample Collection

This study was conducted in four Korean coastal regions representing distinct geographic settings: Jejudo (Seogwipo), Wando, Somaemuldo and Ulleungdo. These regions were selected to provide a standardized comparison across coastal environments that differ in geographic location, coastal configuration and degree of geographic isolation. Jejudo is located in the southernmost part of Korea and is relatively exposed to offshore waters; Wando is situated within a semi‐enclosed coastal area with active aquaculture activities; Somaemuldo is located between coastal and offshore waters; and Ulleungdo is an oceanic island characterized by geographic isolation. These descriptions are based on geographic and regional characteristics and are not intended to represent quantitatively measured environmental conditions.

To evaluate spatial patterns and differences between two seasonal sampling periods in bacterial communities, samples were collected during March and September, representing post‐winter and post‐summer periods, respectively. These sampling periods were selected to provide seasonal snapshots of bacterial community structure and should not be interpreted as representing the full annual seasonal cycle. At each sampling location and sampling period, three field‐replicate surface‐water samples were collected separately using individual sterile sampling bottles. Each replicate represented a separate water collection rather than a subsample divided from a single bulk‐water sample. The replicate samples were filtered separately, and DNA extraction and PCR amplification were performed independently for each sample. Each replicate was subsequently sequenced as a separate library. A total of 24 field‐replicate samples were analysed across the four locations and two sampling periods.

To ensure comparability among regions and minimize the influence of vertical mixing and stratification, all samples were collected from the same surface layer (0–0.5 m depth) using sterile sampling containers. Surface seawater was collected at each station using sterile 2 L polypropylene bottles and transported to the laboratory immediately after collection under dark refrigerated conditions (4°C). Detailed information on sampling coordinates and collection dates is provided in Table S1, and sample IDs are provided in Table S2. To obtain microbial biomass, 1–2 L of seawater per sample was filtered within 24 h of collection using a vacuum filtration system. Filtration volume was adjusted depending on sample turbidity and filtration efficiency. Because filtration volume varied among samples, sequencing data were subsequently normalized by rarefaction prior to diversity and community analyses to minimize potential biases associated with differences in sequencing depth. Seawater was filtered through 0.22 μm pore‐size polyethersulfone (PES) membrane filters (47 mm diameter; Merck Millipore, Germany), which are commonly used for the recovery of free‐living bacterial fractions in marine microbial studies. Following filtration, membrane filters were transferred into sterile tubes and stored at −80°C until DNA extraction.

### 2.2 DNA Extraction and Sequencing

Total genomic DNA was extracted from membrane filters using the DNeasy PowerWater Kit (QIAGEN, Germany) according to the manufacturer's protocol. To improve cell lysis efficiency for mechanically resistant bacterial taxa, an additional bead‐beating step was performed. Filters were processed using a FastPrep‐24 5G instrument (MP Biomedicals, USA) at 6.0 m s^−1^ for 40 s, repeated three times with intermittent ice cooling to minimize DNA damage and thermal degradation.

DNA concentration was measured using a Qubit 4.0 Fluorometer (dsDNA HS Assay Kit; Thermo Fisher Scientific, USA) and DNA purity was evaluated based on A260/A280 and A260/A230 ratios using a NanoDrop One spectrophotometer (Thermo Fisher Scientific, USA). Extracted DNA was stored at −20°C until PCR amplification.

The V4 hypervariable region of the 16S rRNA gene was amplified using the universal primer pair 515F (5′‐GTGYCAGCMGCCGCGGTAA‐3′) and 806R (5′‐GGACTACNVGGGTWTCTAAT‐3′) following the Earth Microbiome Project (EMP) protocol (Caporaso et al. 2012). PCR amplification was performed in 25 μL reaction volumes containing 10 ng of template DNA and KAPA HiFi HotStart ReadyMix (Roche, Switzerland). Thermal cycling conditions consisted of an initial denaturation at 95°C for 3 min, followed by 25 cycles of 95°C for 30 s, 55°C for 30 s and 72°C for 30 s, with a final extension at 72°C for 5 min. Negative controls (no‐template controls) were included in each PCR batch to monitor potential contamination throughout the amplification process. No visible amplification products were observed in any negative‐control reactions. PCR products were purified using Agencourt AMPure XP magnetic beads (Beckman Coulter, USA), quantified and pooled at equimolar concentrations for library preparation. Sequencing libraries were prepared following standard Illumina protocols and sequenced using the Illumina MiSeq platform (MiSeq Reagent Kit v2, 2 × 250 bp paired‐end reads; Illumina, USA). Raw sequence data generated from all samples were subsequently subjected to quality‐control procedures prior to downstream bioinformatic analyses.

### 2.3 Sequence Data Processing and Taxonomic Assignment

Raw paired‐end reads generated on the Illumina MiSeq platform were processed using QIIME 2 (version 2022.11) (Bolyen et al. 2019). Raw read counts ranged from 24,334 to 232,692 per sample and retained read counts after quality filtering ranged from 24,086 to 232,022 (Table S2). Primer sequences were removed prior to analysis, and low‐quality reads were trimmed and filtered based on quality score profiles. Quality‐filtered reads were subsequently merged, and chimera sequences were removed using the consensus method implemented in the vsearch algorithm (Rognes et al. 2016).

Operational taxonomic units (OTUs) were generated through de novo clustering at 97% sequence similarity using vsearch. Although amplicon sequence variant (ASV)‐based approaches are increasingly used in microbial ecology, OTU clustering was selected to maintain consistency with previous Korean coastal bacterial community studies and to facilitate regional community comparisons. The resulting OTU table was used as the basis for all subsequent ecological analyses. To minimize bias caused by differences in sequencing depth among samples, the OTU table was rarefied prior to alpha‐ and beta‐diversity analyses. All samples were generated using the same sequencing platform and processed through a standardized bioinformatic workflow, thereby ensuring comparability among samples. Taxonomic classification of representative OTU sequences was performed using the naïve Bayes classifier implemented in QIIME 2 with the SILVA database (release 138.1) as the reference database (Quast et al. 2013). The database was trimmed to match the V4 region amplified by the 515F/806R primer pair. Community composition analyses were conducted at the phylum, class and genus levels. Species‐level assignments were treated as putative taxonomic assignments because the V4 region does not always provide reliable species‐level resolution for several marine bacterial genera, including *Vibrio*, *Pseudomonas* and *Acinetobacter*.

In this study, the PPB dataset comprised OTUs assigned to 23 genera selected based on taxonomic affiliation and literature‐supported associations with infections in humans or aquatic organisms (Table S3). Candidate PPB genera were compiled with reference to the Enhanced Infectious Diseases Database (Wardeh et al. 2015), the Aquaculture Bacterial Pathogen Database (Lo et al. 2023) and the Human Pathogenic Bacteria Database (Bartlett et al. 2022), and were further screened against relevant peer‐reviewed literature. Genera containing species or strains reported to be associated with opportunistic infection or disease in humans or aquatic organisms were matched against the taxa detected in the OTU dataset. OTUs assigned to the resulting 23 genera were extracted from the total OTU table to construct a separate PPB dataset. For analyses of PPB assemblage composition, PPB abundances were renormalized within the PPB subset so that their relative abundances summed to 100% within each sample. For SIMPER analysis, however, the original PPB relative abundances expressed as percentages of the total bacterial community were used without renormalization within the PPB subset. For taxonomic‐composition summaries in Figures 4D–F and 6, Tables S6D–F and S8, however, PPB abundances were expressed as percentages of the total bacterial community rather than being renormalized within the PPB subset. Accordingly, PPB identification using 16S rRNA gene sequencing does not directly confirm the presence of pathogenic strains, virulence genes, infectivity, ecological function or actual disease risk (Janda and Abbott 2007; Johnson et al. 2019). Rather, these taxa were treated as an ecological grouping for comparing their distributions among the four sampled locations and between the March and September sampling periods.

### 2.4 Alpha‐ and Beta‐Diversity Analyses

Alpha diversity was evaluated using observed OTUs, the Shannon index and the Gini–Simpson index. These indices were calculated separately for the total bacterial community and the putative PPB assemblage. The effects of sampling location, sampling period and their interaction on each alpha‐diversity index were evaluated using Type III two‐way analysis of variance (ANOVA). Gini–Simpson values were logit‐transformed before inferential analysis, whereas observed OTUs and Shannon index values were analysed without transformation. Residual normality and homogeneity of variance were evaluated using the Shapiro–Wilk and Levene's tests, respectively. Statistical significance was defined as *p* < 0.05. No post hoc pairwise comparisons were included in the revised inferential analysis; therefore, multiple‐comparison adjustment was not applicable to the reported alpha‐diversity results.

Beta diversity was evaluated based on Bray–Curtis dissimilarity calculated from relative abundance data (Bray and Curtis 1957). Regional and seasonal differences in community composition were visualized using principal coordinates analysis (PCoA) based on Bray–Curtis dissimilarity matrices. Prior to PERMANOVA, homogeneity of multivariate dispersion among groups was evaluated using the betadisper function in the vegan package to verify that differences in community composition were not primarily attributable to differences in within‐group dispersion. To evaluate the effects of region, season and their interaction (Region × Season) on community structure, permutational multivariate analysis of variance (PERMANOVA) based on Bray–Curtis distances was performed (Anderson 2001). The analysis model was specified as ‘Community dissimilarity ~ Region × Season’ and implemented using the adonis2 function in the vegan package (Oksanen et al. 2022) in R with 9999 permutations. Model terms were evaluated sequentially. Because the sampling location × sampling period interaction was significant, follow‐up simple‐effect analyses were conducted to test sampling‐location effects within each sampling period and sampling‐period effects within each location. Pairwise PERMANOVA comparisons were subsequently conducted among locations within each sampling period and between sampling periods within each location. *p*‐Values from the follow‐up analyses were adjusted using the Benjamini–Hochberg procedure (Benjamini and Hochberg 1995). Multivariate dispersion was evaluated separately by sampling location, sampling period and their combined groups.

The three separately collected water samples at each location and sampling period were treated as field replicates and as independent experimental units in all statistical analyses. Analyses of the total bacterial community and the PPB assemblage were conducted separately.

### 2.5 Similarity Percentage (SIMPER) and Indicator Species Analyses

SIMPER analysis was performed based on Bray–Curtis dissimilarity to identify taxa contributing substantially to differences in PPB assemblages among regions (Clarke 1993). Pairwise comparisons among regions were conducted using genus‐level and putative species‐level PPB abundances expressed as percentages of the total bacterial community. These abundances were not renormalized to 100% within the PPB subset prior to SIMPER analysis. The relative importance of each taxon in contributing to observed community dissimilarity was evaluated based on its contribution to overall dissimilarity. Taxa were ranked according to their contribution to total community dissimilarity and cumulative contributions were calculated. Because SIMPER is a descriptive method, taxa identified as major contributors were interpreted as contributors to compositional differences among sampling locations rather than as ecological drivers of community variation.

Indicator species analysis was conducted using the multilevel pattern analysis implemented in the multipatt function of the indicspecies package in R. Associations with individual sampling locations and combinations of up to three locations were evaluated using the IndVal.g statistic. Statistical significance was assessed using 9999 permutations. *p*‐Values obtained for all 181 tested taxa were adjusted using the Benjamini–Hochberg procedure (Benjamini and Hochberg 1995) and taxa with BH‐adjusted *p* < 0.05 were considered statistically significant indicator taxa. Indicator taxa were interpreted only as taxa statistically associated with particular sampling locations or combinations of locations. Because taxonomic assignments were based on the 16S rRNA gene V4 region, these associations were not interpreted as direct evidence of pathogenic potential, virulence, disease risk or ecological function.

### 2.6 Software and Reproducibility

All statistical analyses and visualizations were performed using R (version 4.2.2). Beta‐diversity calculations, PERMANOVA, multivariate dispersion tests (betadisper) and SIMPER analyses were conducted using the vegan package, whereas indicator species analysis was performed using the indicspecies package. Figures were generated using the ggplot2 package. Sequence processing, including quality filtering, OTU clustering and taxonomic assignment, was conducted in QIIME 2 (version 2022.11) following a standard workflow for 16S rRNA gene amplicon sequencing data (Bolyen et al. 2019). Key analytical parameters, including OTU clustering threshold, permutation number and criteria used to define PPB, are provided in the main text and Supplementary Material. All analyses were performed using reproducible workflows and fixed analytical parameters across samples to ensure consistency in regional comparisons.

## 3 Results

### 3.1 Overview of Study Regions and Sampling

In this study, bacterial communities were investigated across four Korean coastal regions representing distinct geographic settings: Jejudo (Seogwipo), Wando, Somaemuldo and Ulleungdo (Figure 1). Surface seawater samples were collected from the same surface layer during March and September, resulting in a total of 24 samples. The study regions were distributed across latitudes ranging from 33.22° N to 37.53° N and longitudes ranging from 126.56° E to 130.81° E (Table S1).

**FIGURE 1:**
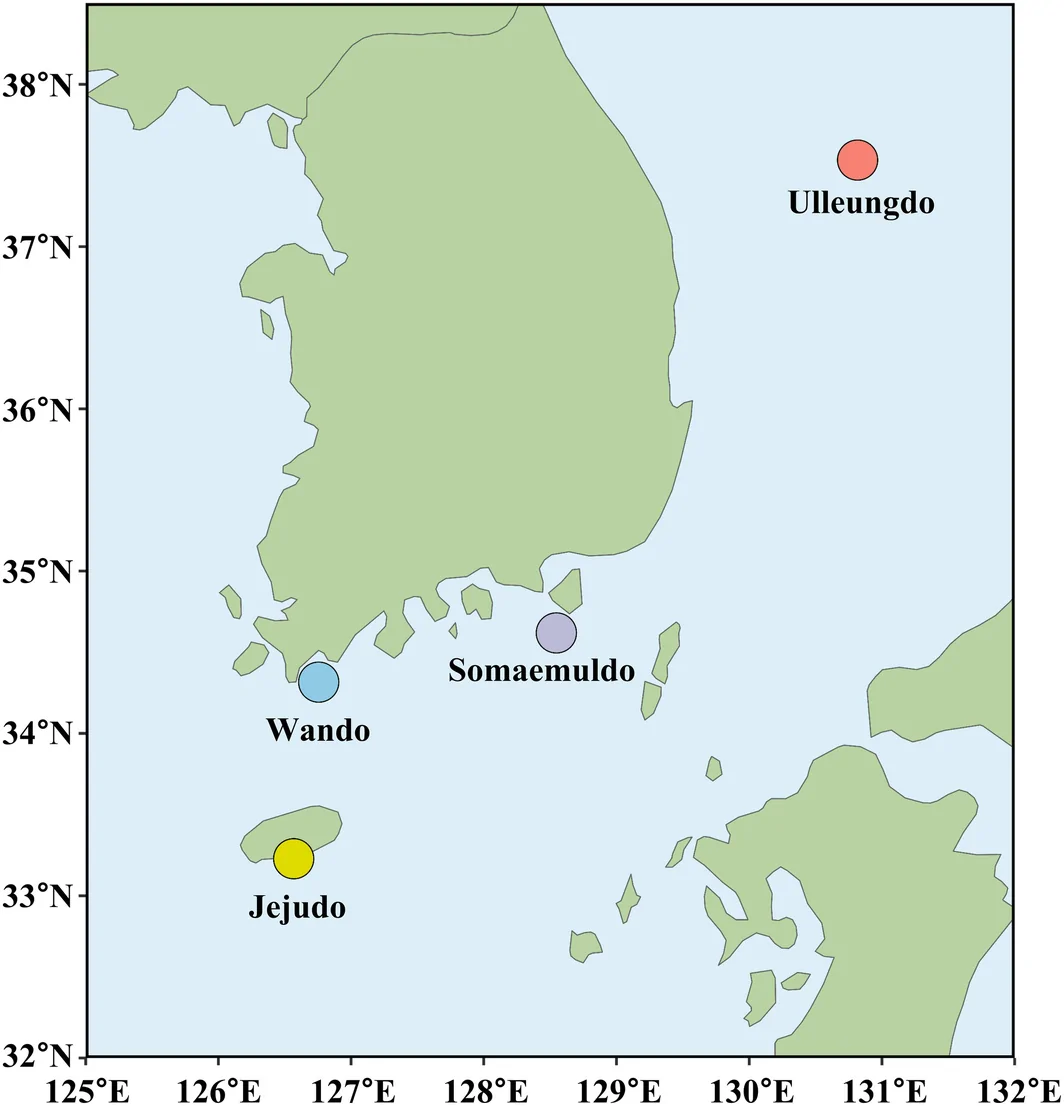
Geographical locations of the four Korean coastal sampling locations.

### 3.2 Alpha‐Diversity Patterns

Alpha‐diversity indices of the total bacterial community exhibited location‐ and sampling‐period‐associated variation (Figure 2A–C; Table S4A). The highest observed OTU richness was recorded at Ulleungdo in September (266.3 ± 10.0), whereas the lowest value was recorded at Wando in March (100.7 ± 20.6). Shannon and Gini–Simpson diversity were also highest at Ulleungdo in September (4.99 ± 0.06 and 0.982 ± 0.003, respectively) and lowest at Wando in March (2.47 ± 0.73 and 0.683 ± 0.199, respectively). Type III two‐way ANOVA detected significant effects of sampling location and sampling period on observed OTUs, Shannon diversity and Gini–Simpson diversity (Table S4B). Significant sampling location × sampling period interactions were detected for observed OTUs (*F*_3,16_ = 87.03, *p* < 0.001) and Shannon diversity (*F*_3,16_ = 4.74, *p* = 0.015), whereas the interaction was not significant for Gini–Simpson diversity (*F*_3,16_ = 2.86, *p* = 0.070). These interaction effects indicate that location‐associated patterns in observed OTUs and Shannon diversity differed between the March and September sampling periods.

**FIGURE 2:**
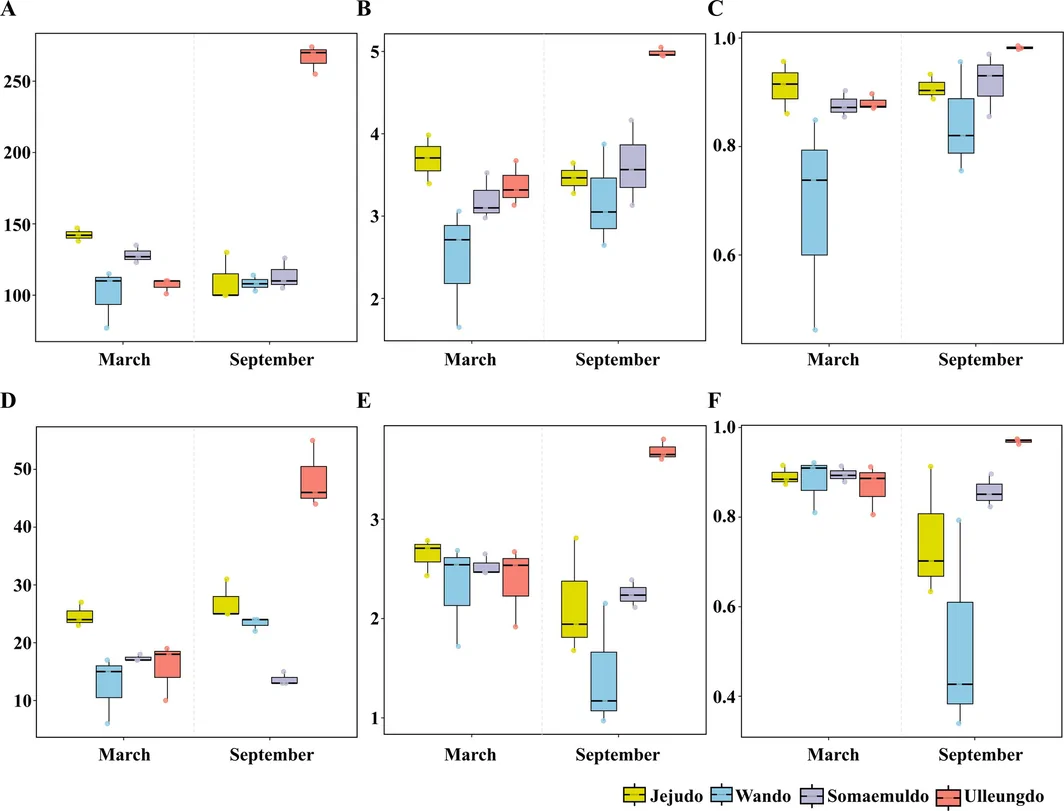
Alpha‐diversity patterns of the total bacterial community and putative pathogen‐associated bacterial taxa (PPB) assemblage across four sampling locations and two sampling periods. (A–C) Observed OTUs, Shannon index and Gini–Simpson index for the total bacterial community. (D–F) Corresponding alpha‐diversity indices for the PPB assemblage. Boxes represent the interquartile range, horizontal lines indicate medians and points represent individual field replicates.

Alpha diversity of the PPB assemblage was lower in absolute magnitude than that of the total bacterial community and also exhibited variation among sampling locations and between sampling periods (Figure 2D–F; Table S4A). The highest observed OTU richness, Shannon diversity and Gini–Simpson diversity were recorded at Ulleungdo in September (48.3 ± 5.9, 3.69 ± 0.10 and 0.970 ± 0.006, respectively). The lowest observed OTU richness occurred at Wando in March (12.7 ± 5.9), whereas the lowest Shannon and Gini–Simpson values occurred at Wando in September (1.43 ± 0.63 and 0.520 ± 0.241, respectively). Sampling location significantly affected all three PPB alpha‐diversity indices (all *p* ≤ 0.002; Table S4B). The sampling‐period effect was significant for observed OTUs (*F*_1,16_ = 47.11, *p* < 0.001), but not for Shannon diversity (*F*_1,16_ = 0.29, *p* = 0.596) or Gini–Simpson diversity (*F*_1,16_ = 2.77, *p* = 0.115). Significant sampling location × sampling period interactions were detected for observed OTUs, Shannon diversity and Gini–Simpson diversity (all *p* ≤ 0.001), indicating that sampling‐period‐associated patterns differed among locations. No significant violations of residual normality or homogeneity of variance were detected for any alpha‐diversity index (all Shapiro–Wilk and Levene's test *p*‐values > 0.05).

### 3.3 Beta Diversity and Community Structure

PCoA revealed differences in community structure among sampling locations and between sampling periods for both the total bacterial community and the PPB assemblage (Figure 3). For the total bacterial community, PCoA1 and PCoA2 explained 25.2% and 19.7% of the total variance, respectively (Figure 3A). Field replicates from the same sampling location and sampling period generally clustered closely together, while differences between March and September were apparent within several locations. The relative positions of samples also indicated location‐associated variation in total bacterial community composition. For the PPB assemblage, PCoA1 and PCoA2 explained 37.2% and 22.6% of the total variance, respectively (Figure 3B). Field replicates generally showed similar ordination patterns within each sampling location and period. Differences between March and September were apparent across the sampled locations, with the separation between the two sampling periods being particularly evident for Ulleungdo. Overall, the PCoA patterns indicated that both sampling location and sampling period were associated with variation in PPB assemblage composition. Multivariate dispersion did not differ significantly by sampling location, sampling period or their combined groups for the total bacterial community (all BH‐adjusted *p* ≥ 0.468; Table S5C). For the PPB assemblage, dispersion did not differ significantly by sampling period or combined sampling location × sampling period groups, whereas a significant difference was detected among sampling locations (pseudo‐*F* = 6.45, BH‐adjusted *p* = 0.012). Therefore, the significant sampling‐location effect detected by PERMANOVA for the PPB assemblage should be interpreted cautiously because it may partly reflect differences in within‐location dispersion.

**FIGURE 3:**
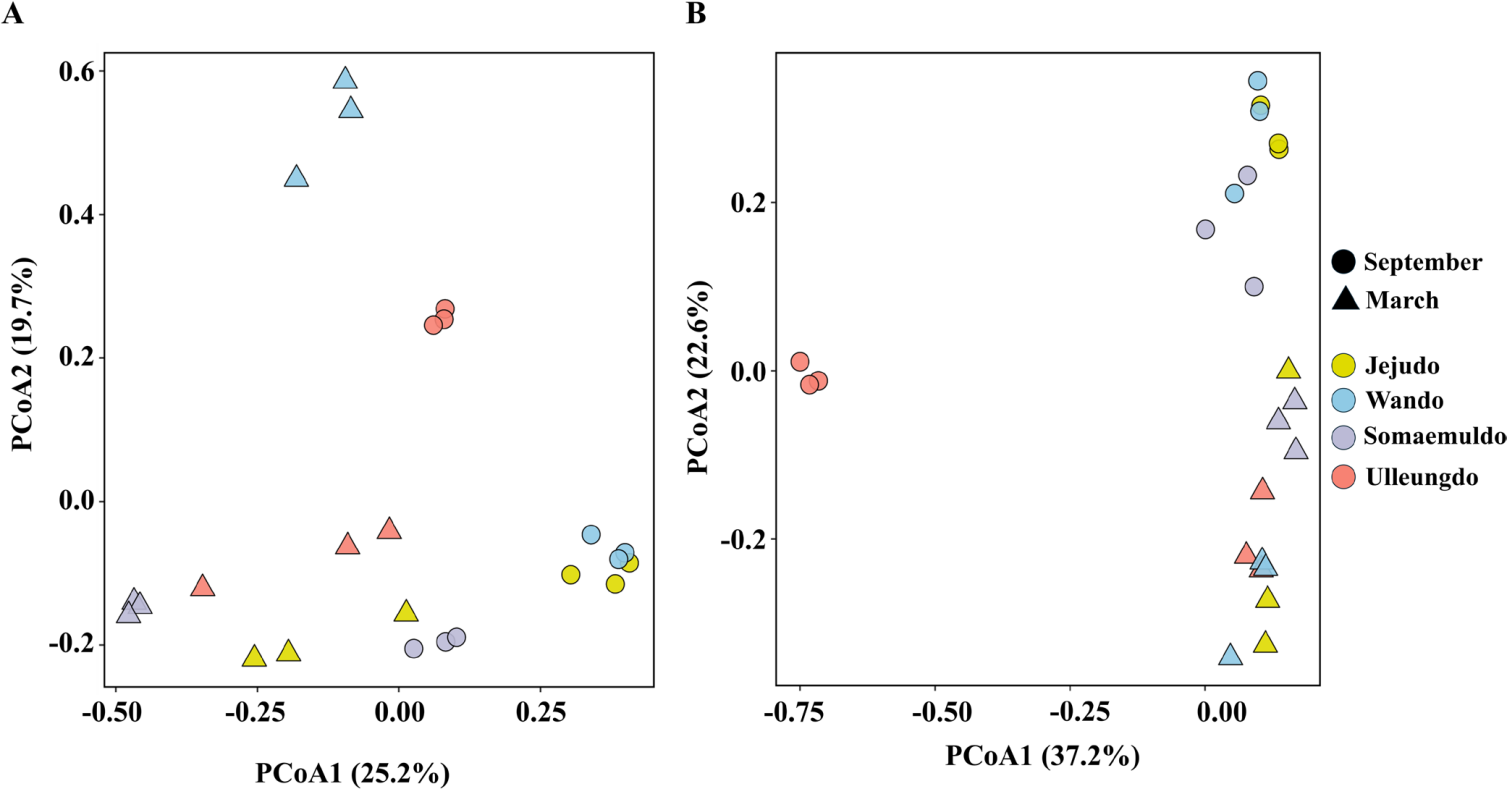
Beta diversity and community structure of total bacterial communities and PPB assemblages based on principal coordinates analysis (PCoA). PCoA ordinations derived from Bray–Curtis dissimilarity matrices for (A) total bacterial communities and (B) PPB assemblages. Each point represents an individual field replicate (*n* = 24). Colours indicate sampling locations and shapes indicate sampling periods (March and September).

PERMANOVA showed significant effects of sampling location, sampling period and their interaction on the composition of the total bacterial community (Table S5A). Sampling location explained 35.7% of the total variation (*R* ^2^ = 0.357, pseudo‐*F* = 13.12, *p* < 0.001), sampling period explained 20.9% (*R* ^2^ = 0.209, pseudo‐*F* = 23.06, *p* < 0.001) and their interaction explained 28.9% (*R* ^2^ = 0.289, pseudo‐*F* = 10.61, *p* < 0.001). Residual variation accounted for the remaining 14.5% of the total variation. The PPB assemblage was also significantly associated with sampling location, sampling period and their interaction (Table S5A). Sampling location explained 27.3% of the variation (*R* ^2^ = 0.273, pseudo‐*F* = 7.37, *p* < 0.001), sampling period explained 23.1% (*R* ^2^ = 0.231, pseudo‐*F* = 18.70, *p* < 0.001) and their interaction explained 29.8% (*R* ^2^ = 0.298, pseudo‐*F* = 8.02, *p* < 0.001). Residual variation accounted for the remaining 19.8% of the total variation.

Because the interaction between sampling location and sampling period was significant, follow‐up analyses were conducted separately within each sampling period and location (Table S5B). Community composition differed significantly among the four sampled locations in both March and September for the total bacterial community (March: *R* ^2^ = 0.776, pseudo‐*F* = 9.25, adjusted *p* < 0.001; September: *R* ^2^ = 0.857, pseudo‐*F* = 15.96, adjusted *p* < 0.001) and the PPB assemblage (March: *R* ^2^ = 0.500, pseudo‐*F* = 2.66, adjusted *p* < 0.001; September: *R* ^2^ = 0.844, pseudo‐*F* = 14.47, adjusted *p* < 0.001). However, none of the individual pairwise comparisons among locations within each sampling period or between sampling periods within each location was statistically significant after multiple‐testing correction (all BH‐adjusted *p* = 0.10). The resolution of these pairwise permutation tests was limited by the availability of only three field replicates per group.

### 3.4 Taxonomic Composition of Total Bacterial Communities

The taxonomic composition of the total bacterial community differed among regions at the phylum, class and genus levels, and differences between March and September were also observed for several taxa (Figure 4A–C). At the phylum level, Pseudomonadota (formerly Proteobacteria) dominated across all sampled locations and sampling periods (Figure 4A). Wando maintained relative abundances of Pseudomonadota exceeding 90% throughout both sampling periods, while Somaemuldo also exhibited consistently high relative abundances (March: 93.98%; September: 87.56%) (Table S6A). In contrast, Ulleungdo exhibited lower relative abundances of Pseudomonadota during September, whereas Actinomycetota (21.32%) and Bacteroidota (12.39%) accounted for a larger proportion of the community. At Jejudo, the relative abundance of Bacteroidota was higher in September (5.34%) than in March (1.87%).

**FIGURE 4:**
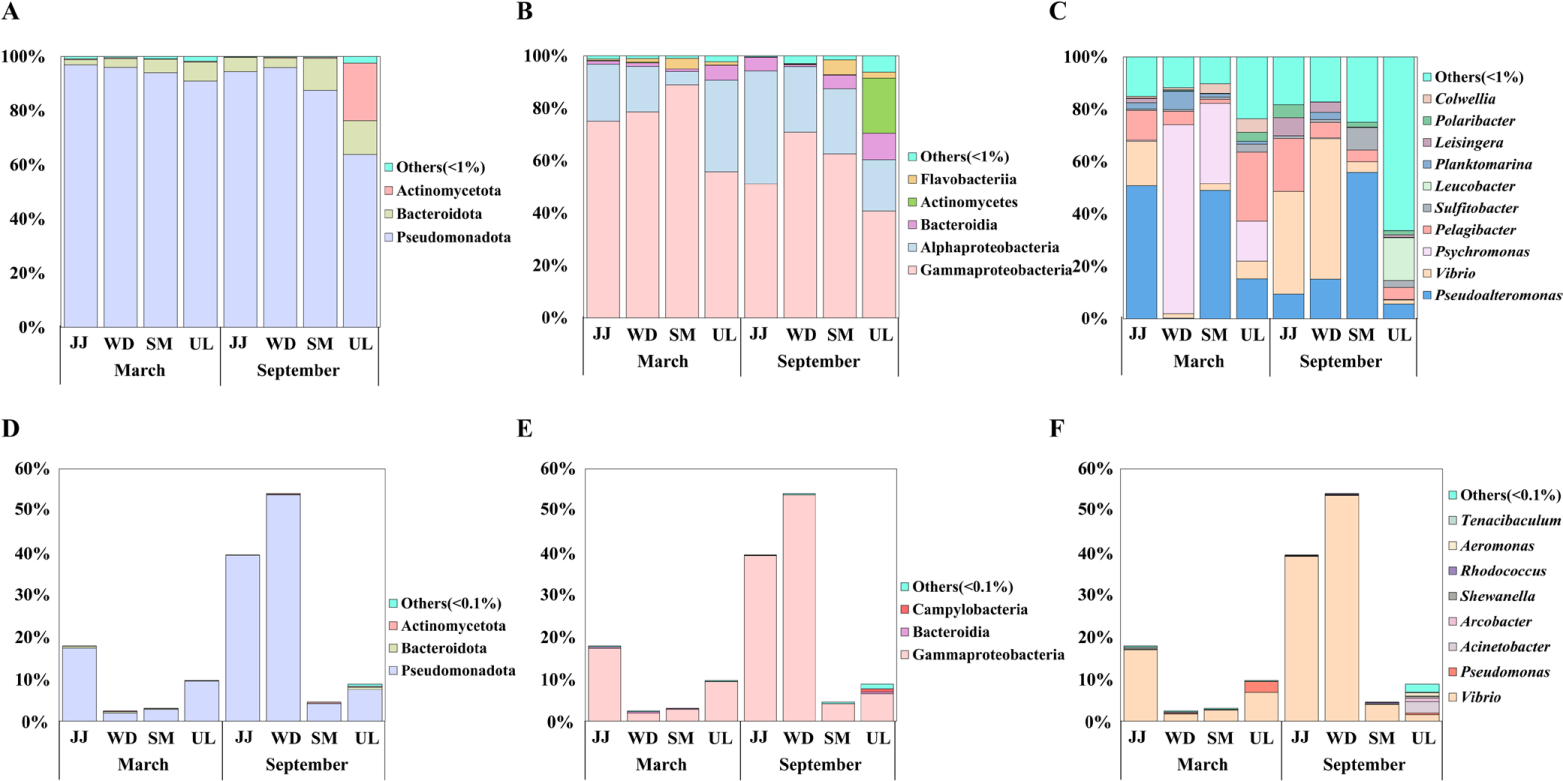
Taxonomic composition of total bacterial communities and PPB assemblages at phylum, class and genus levels. Relative abundance (%) of dominant taxa in total bacterial communities (A–C) and PPB assemblages (D–F) at the phylum (A, D), class (B, E) and genus (C, F) levels. PPB relative abundances in (D–F) are expressed as percentages of the total bacterial community.

At the class level, Gammaproteobacteria and Alphaproteobacteria were the dominant bacterial groups (Figure 4B). Gammaproteobacteria dominated in Jejudo, Wando and Somaemuldo during both sampling periods, with particularly high relative abundances observed at Wando (March: 78.65%; September: 70.90%) (Table S6B). In Ulleungdo, Gammaproteobacteria (55.71%) and Alphaproteobacteria (35.00%) co‐dominated during March, whereas September samples exhibited a lower relative abundance of Gammaproteobacteria and a greater proportional representation of Actinomycetes (20.94%) and Bacteroidia (10.12%).

At the genus level, location‐associated differences in community composition became more pronounced (Figure 4C). The relative abundance of *Vibrio* was higher in September than in March in both Wando and Jejudo. In Wando, *Vibrio* accounted for 53.69% of the community during September, whereas Jejudo exhibited a relative abundance of 39.16% during the same period. *Pseudoalteromonas* dominated in Somaemuldo during both March (48.98%) and September (55.86%) (Table S6C). In Ulleungdo, *Psychromonas* (15.32%) and *Pelagibacter* (26.39%) were major taxa during March, whereas *Leucobacter* represented 16.32% of the community during September. In addition, *Sulfitobacter* exhibited relatively high relative abundances in September at Somaemuldo (8.61%) and in March at Ulleungdo (2.99%).

### 3.5 Taxonomic Composition of the PPB Assemblage

The taxonomic composition of the PPB assemblage also differed among regions at the phylum, class and genus levels (Figure 4D–F). At the phylum level, Pseudomonadota dominated across most regions and sampling periods (Figure 4D). In Wando, the relative abundance of Pseudomonadota was higher in September (53.84%) than in March (1.88%) (Table S6D). A similar pattern was observed in Jejudo, where the relative abundance was 39.43% in September and 17.37% in March. In contrast, Ulleungdo exhibited relatively low relative abundances of Pseudomonadota during September, whereas Bacteroidota (0.53%) and Actinomycetota (0.21%) were also detected.

At the class level, Gammaproteobacteria was the dominant class across all sampled locations (Figure 4E). September samples from Wando (53.83%) and Jejudo (39.34%) exhibited higher relative abundances than March samples (Table S6E). In Ulleungdo, Gammaproteobacteria accounted for 6.48% during September, whereas Bacteroidia (0.42%) and Campylobacteria (0.84%) were also detected.

At the genus level, distinct location‐associated differences in composition were observed (Figure 4F). *Vibrio* was detected in all regions; however, its relative abundance and seasonal distribution patterns differed among regions. In Wando, *Vibrio* accounted for 53.69% of the total bacterial community in September compared with 1.67% in March (Table S6F), whereas in Jejudo it represented 39.16% of the assemblage during September. In contrast, *Vibrio* exhibited relatively lower relative abundances in Ulleungdo and Somaemuldo, where multiple genera co‐occurred. September samples from Ulleungdo contained *Acinetobacter* (2.75%), *Arcobacter* (0.84%), *Aeromonas* (0.77%) and *Pseudomonas* (0.40%). *Tenacibaculum* was detected at low relative abundances in March samples from Jejudo (0.29%) and Wando (0.26%), as well as in September samples from Ulleungdo (0.13%).

### 3.6 SIMPER Analysis

SIMPER analysis based on Bray–Curtis dissimilarity indicated that a limited number of genera contributed substantially to observed differences among PPB assemblages across regions (Table S7). Across all pairwise regional comparisons, *Vibrio* exhibited the highest contribution values. In the Jejudo–Wando comparison, *Vibrio* accounted for a contribution of 0.59 (mean relative abundance: Jejudo 28.06%, Wando 27.68%). In the Jejudo–Ulleungdo comparison, *Vibrio* exhibited the highest single‐genus contribution (0.54; Jejudo 28.06%, Ulleungdo 4.16%). *Vibrio* also represented one of the largest contributors in the Wando–Somaemuldo comparison (0.64).

*Pseudomonas*, *Acinetobacter*, *Arcobacter* and *Aeromonas* also exhibited relatively high contributions depending on regional combinations, particularly in comparisons involving Ulleungdo. In the Somaemuldo–Ulleungdo comparison, *Acinetobacter* (0.11) and *Pseudomonas* (0.10) contributed alongside *Vibrio* (0.21). In the Ulleungdo–Wando comparison, *Acinetobacter* (0.07) and *Pseudomonas* (0.07) likewise contributed to observed dissimilarities. *Tenacibaculum* exhibited relatively higher contributions in comparisons involving Wando (Jejudo–Wando: 0.01; Somaemuldo–Wando: 0.02; Ulleungdo–Wando: 0.01).

Visualization of putative species‐level contribution patterns (Figure 5) showed that OTUs assigned to *Vibrio ordalii* exhibited the highest individual contribution in several regional comparisons, particularly JJ versus WD, WD versus SM and WD versus UL. OTUs assigned to *Vibrio kanaloae* also exhibited relatively high contributions across multiple regional combinations. Comparisons involving Ulleungdo showed notable contributions from OTUs assigned to *Acinetobacter parvus* , *Pseudomonas pelagia* and *Aeromonas* spp. Cumulative contribution plots indicated that a small number of dominant taxa accounted for a large proportion of the observed dissimilarity, whereas a greater number of taxa contributed to cumulative dissimilarity in comparisons involving Ulleungdo and Somaemuldo.

**FIGURE 5:**
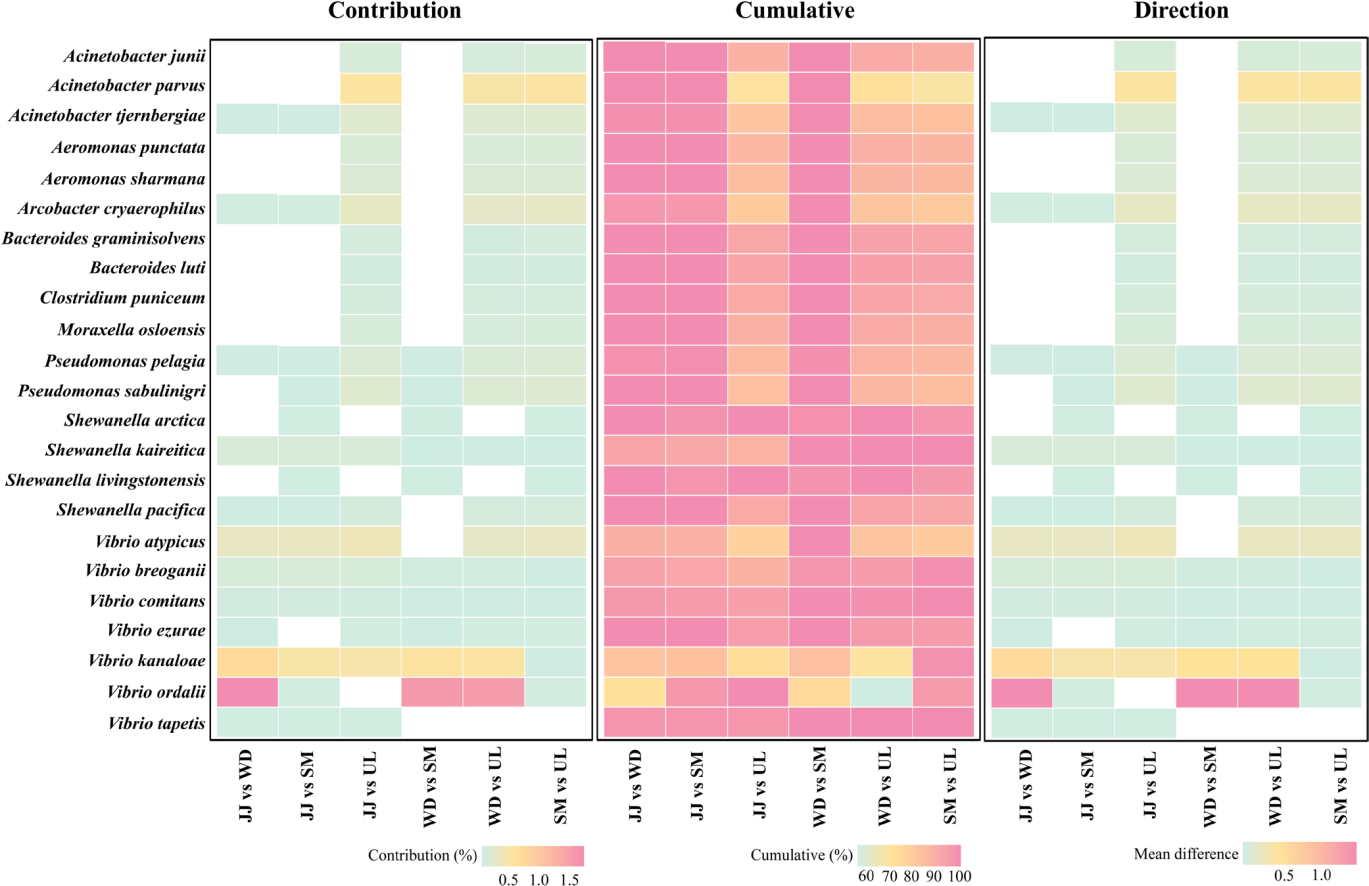
Putative species‐level contributions of PPB taxa to community dissimilarity among the four sampled locations based on SIMPER analysis. The Contribution panel shows the contribution of each taxon to Bray–Curtis dissimilarity for each pairwise comparison, the Cumulative panel shows the cumulative contribution and the Direction panel shows the mean abundance difference between the two locations in each comparison. White cells in the Contribution and Direction panels indicate zero values in the corresponding data matrices and do not represent missing or omitted cells.

### 3.7 Putative Species‐Level Distribution of PPB Taxa

Putative species‐level taxonomic assignments revealed distinct dominant species compositions and relative abundance patterns within PPB assemblages among regions (Figure 6; Table S8). Only taxa with relative abundances ≥ 0.1% in at least one region‐season combination were included in the analysis. Because species‐level classification was inferred from the 16S rRNA gene V4 region, species names presented below should be interpreted as putative taxonomic assignments. Most dominant taxa were assigned to the genus *Vibrio*; however, the relative abundance and composition of individual putative species‐level taxa differed among regions. In Jejudo, multiple *Vibrio*‐associated taxa with relatively high abundances were co‐detected.

**FIGURE 6:**
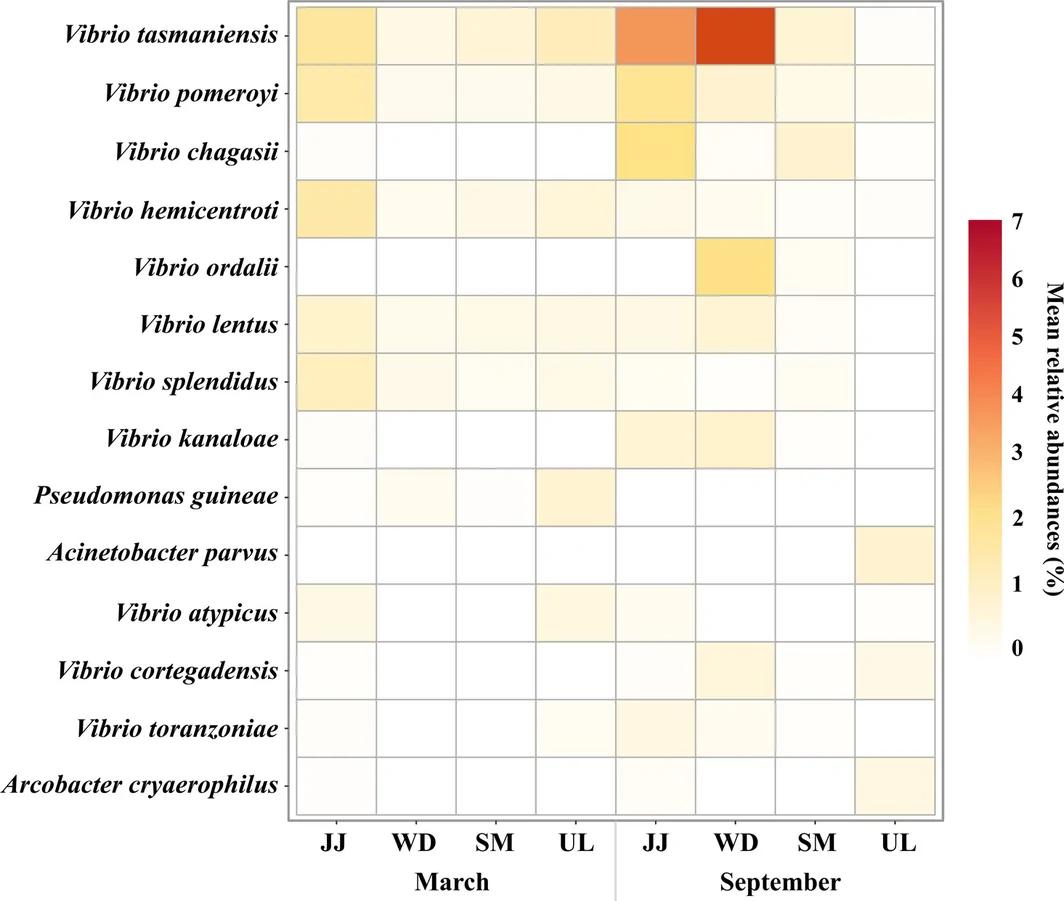
Putative species‐level distributions of PPB taxa at four Korean coastal locations during the March and September sampling periods.

OTUs assigned to *Vibrio tasmaniensis* accounted for 2.07% in March and 4.93% in September in Jejudo, while reaching 6.42% in September at Wando. OTUs assigned to *Vibrio pomeroyi* accounted for 1.86% in March and 2.16% in September in Jejudo but remained below 1% in most other regions. Additional *Vibrio*‐associated taxa detected in Jejudo included OTUs assigned to *Vibrio chagasii* (2.37% in September), *Vibrio hemicentroti* (1.88% in March) and *Vibrio lentus* (1.34% in March). In Wando, OTUs assigned to *V. ordalii* (2.35% in September) and *V. kanaloae* (1.36% in September) exhibited relatively high relative abundances. In contrast, most *Vibrio*‐associated taxa detected in Somaemuldo and Ulleungdo exhibited relative abundances below 1%.

Several non‐*Vibrio* taxa were detected only in specific regions. OTUs assigned to *Pseudomonas guineae* accounted for 1.25% in March samples from Ulleungdo, whereas OTUs assigned to *A. parvus* reached 1.29% in September samples from Ulleungdo.

### 3.8 Indicator Species Analysis

Indicator species analysis evaluated the associations of 181 PPB taxa with individual sampling locations and combinations of up to three locations. Based on permutation tests with 9999 permutations, 24 taxa showed nominal associations with raw *p*‐values below 0.05. However, none remained statistically significant after Benjamini–Hochberg correction for multiple testing (minimum BH‐adjusted *p* = 0.181; Table S9). Therefore, no statistically significant sampling‐location‐associated indicator taxa were detected in the PPB assemblage under the FDR‐controlled significance criterion.

## 4 Discussion

### 4.1 Spatial Differentiation of Coastal Bacterial Communities

This study demonstrated clear spatial differentiation of surface‐water bacterial communities across four Korean coastal regions representing distinct geographic settings. Both the total bacterial community and the putative PPB assemblage exhibited differences in alpha diversity, beta diversity and taxonomic composition among the four sampled coastal locations (Figures 2, 3, 4), indicating location‐associated heterogeneity in microbial assemblages under the conditions examined in this study.

Coastal environments are often characterized by substantial spatial heterogeneity resulting from interactions among multiple physical, chemical and biological processes (Cloern et al. 2016; Logares et al. 2020). The four regions examined in this study were selected to represent geographically distinct coastal settings within Korean waters. Because concurrent environmental metadata were not collected, the mechanisms underlying the observed location‐associated differences cannot be directly evaluated. Therefore, the present results are interpreted primarily as evidence of spatial community structuring rather than as evidence for specific environmental drivers. Importantly, despite all samples being collected from the same surface layer using a standardized sampling and analytical framework, distinct regional community patterns were consistently observed. This finding suggests that bacterial community composition differs among Korean coastal regions and that these differences remain detectable across multiple sampling periods.

Previous global marine microbiome studies have demonstrated that microbial communities often exhibit strong spatial structuring across environmental and geographic gradients (Sunagawa et al. 2015; Logares et al. 2020). Although the present study does not directly evaluate the environmental processes responsible for these patterns, the observed location‐associated differences indicate that distinct microbial community baselines may exist among Korean coastal regions. Ulleungdo exhibited community compositions that differed from those of the other regions, whereas Wando, Jejudo and Somaemuldo displayed distinct compositional patterns of their own. These location‐associated differences provide a useful microbial baseline for future monitoring studies that incorporate environmental metadata and long‐term observations.

### 4.2 Interaction Between Sampling Location and Sampling Period

PCoA results indicated that Korean coastal bacterial communities were organized around region‐specific community structures (regional baselines), while differences between March and September were observed within these regional frameworks (Figure 3). Samples from the same region were positioned closely together in ordination space, indicating relatively consistent regional community organization. Visual differences between the March and September samples were apparent within several sampled locations; however, individual within‐location comparisons were not statistically significant after multiple‐testing correction. Thus, the ordination patterns should be interpreted as descriptive differences between the two sampling periods rather than as evidence of comprehensive seasonal dynamics.

Ulleungdo samples showed a distinct ordination pattern relative to the other sampled locations, with differences also apparent between the March and September sampling periods. PERMANOVA showed significant effects of sampling location, sampling period and their interaction on total bacterial community composition (Table S5A). Sampling location explained 35.7% of the variation, sampling period explained 20.9% and their interaction explained 28.9%. The significant interaction indicates that differences between the March and September sampling periods were not consistent across all locations. Follow‐up analyses confirmed significant differences in community composition among the four sampled locations within both sampling periods, although no individual pairwise comparison remained significant after multiple‐testing correction (Table S5B).

Overall, the observed community patterns reflected differences among the four sampled coastal locations and between the March and September sampling periods, with the magnitude of sampling‐period‐associated differences varying among locations. These findings provide preliminary location‐specific information that may support future assessments incorporating broader spatial replication and repeated seasonal sampling.

### 4.3 Taxonomic Composition of the Total Bacterial Community

Bacterial communities across the four Korean coastal regions shared a common taxonomic foundation at the phylum level, with Pseudomonadota dominating in all regions (Figure 4A). This pattern is consistent with previous global marine microbiome studies reporting that upper‐level taxonomic structures are relatively conserved in surface marine microbial communities (Sunagawa et al. 2015; Logares et al. 2020). These findings suggest that Korean coastal waters are also embedded within the broader framework of marine microbial biogeography.

In contrast, location‐associated differences became more pronounced at finer taxonomic resolutions, particularly at the class and genus levels (Figure 4B,C). Thus, regional community differentiation appeared to be associated primarily with shifts in relative abundances at the class and genus levels rather than by turnover of dominant phyla. Alphaproteobacteria and Gammaproteobacteria were the dominant classes across all sampled locations, although their relative abundances varied among regions (Table S6B).

At the genus level, differences in relative abundance between dominant taxa such as *Pelagibacter*, *Vibrio*, *Psychromonas* and *Pseudoalteromonas* became even more apparent (Figure 4C). These results indicate that regional differentiation was more pronounced at lower taxonomic levels than at the phylum level. Because environmental variables were not measured concurrently, the ecological processes underlying these patterns cannot be directly evaluated. Nevertheless, the observed differences demonstrate that geographically distinct Korean coastal regions support different bacterial community compositions and relative abundance structures. Together, these findings contribute to the establishment of a standardized preliminary, location‐specific baseline information for Korean coastal waters, providing a valuable reference for future microbial monitoring, environmental assessments and studies integrating concurrent environmental measurements.

### 4.4 Spatial Differentiation of the PPB Assemblage

In coastal environments, PPB assemblages provide useful information for understanding patterns of microbial community organization and spatial variation. The widespread occurrence of *Vibrio* across all sampled locations (Figure 4F) is consistent with previous studies reporting that *Vibrio* is commonly detected in marine environments (Baker‐Austin et al. 2017; Trinanes and Martinez‐Urtaza 2021). However, the present results indicate that location‐associated differences were not associated solely with the occurrence of *Vibrio*, but also with variation in the relative composition of multiple bacterial genera.

SIMPER analysis revealed that *Vibrio* showed the highest contribution to Bray–Curtis dissimilarity across all regional comparisons (0.21–0.64; Table S7). Because SIMPER is a descriptive approach, these results should be interpreted as indicating taxa contributing to observed compositional differences rather than ecological drivers of location‐associated variation. Taxa such as *Pseudomonas*, *Acinetobacter*, *Arcobacter*, *Aeromonas*, *Shewanella* and *Tenacibaculum* also exhibited relatively high contributions depending on the regional comparison (Figure 5; Table S7). Notably, the cumulative contribution of the top five to six genera reached approximately 0.70 in most regional comparisons, suggesting that observed location‐associated differences in PPB assemblages were associated with variation in the relative abundances of a limited number of dominant taxa.

Ulleungdo and Somaemuldo exhibited PPB assemblages that differed from those observed in the other regions. In Ulleungdo, *Acinetobacter* and *Pseudomonas* exhibited relatively high contributions, particularly in comparisons with Somaemuldo (0.11 and 0.10, respectively) and Wando (0.07 and 0.07, respectively) (Table S7). However, because environmental variables such as organic matter concentration, particle abundance and hydrographic conditions were not measured in this study, the ecological mechanisms underlying these patterns cannot be directly evaluated. Somaemuldo exhibited an intermediate assemblage structure in which multiple dominant taxa co‐occurred. Together, these results indicate that PPB assemblage composition differed among the four sampled coastal locations. However, multivariate dispersion also differed significantly among sampling locations for the PPB assemblage (Table S5C). Therefore, the significant sampling‐location effect detected by PERMANOVA should be interpreted cautiously because it may partly reflect differences in within‐location dispersion as well as differences in assemblage composition. The PPB assemblage examined in this study represents an ecological grouping based on taxonomic affiliation and literature‐supported associations. Accordingly, these results should not be interpreted as direct evidence of pathogenic strains, virulence potential, ecological function or disease risk.

### 4.5 Putative Species‐Level Spatial Distribution Patterns

Putative species‐level taxonomic assignment provided a finer‐resolution description of spatial variation within the PPB assemblage than analyses conducted at the genus level. Because species‐level classifications were inferred from the 16S rRNA gene V4 region, the taxa discussed below should be interpreted as putative taxonomic assignments. Although similar dominant patterns were observed among regions at higher taxonomic levels, relative abundance and occurrence frequency at the putative species level exhibited region‐specific patterns (Figure 6).

The observed putative species‐level distribution patterns could be broadly categorized into three groups: (1) taxa exhibiting relatively high abundances within specific regions, such as OTUs assigned to *V. ordalii* in Wando; (2) taxa occurring across multiple regions but differing in relative abundance, such as OTUs assigned to *V. tasmaniensis* and (3) taxa detected only in a limited number of regions, such as OTUs assigned to *A. parvus* in Ulleungdo (Table S8). Together, these patterns contributed to differences in community composition among regions at the putative species level (Figure 6; Martiny et al. 2006; Logares et al. 2020).

Rather than directly evaluating the pathogenicity of individual taxa, this study focused on spatial variation within the PPB assemblage as a whole (Table S8). Notably, even in the absence of comprehensive environmental metadata, inter‐regional differences were maintained more consistently than differences between the March and September sampling periods. These results support the presence of location‐associated variation in PPB assemblage composition at the four sampled Korean coastal locations and provide additional baseline information at finer taxonomic resolution.

### 4.6 Indicator Species Analysis

Indicator species analysis evaluates the strength of association between individual taxa and particular sampling groups based on taxon specificity and fidelity (Dufrêne and Legendre 1997; De Cáceres and Legendre 2009). In the present study, 24 of the 181 tested PPB taxa showed nominal associations with individual sampling locations or combinations of locations based on raw *p*‐values below 0.05. However, none remained statistically significant after Benjamini–Hochberg correction for multiple testing (minimum BH‐adjusted *p* = 0.181; Table S9). Therefore, no statistically supported sampling‐location‐associated indicator taxa were identified under the applied FDR‐controlled significance criterion.

The 24 nominal associations reported in Table S9 should consequently be regarded as exploratory observations rather than evidence of location‐specific indicator taxa. Furthermore, because taxonomic assignments were inferred from the 16S rRNA gene V4 region, the reported taxonomic identities should be considered putative and do not provide direct evidence of pathogenic potential, virulence, disease risk or ecological function.

### 4.7 Study Limitations and Ecological Implications

A major limitation of this study is the lack of sufficient concurrent environmental metadata, including temperature, salinity, nutrients and chlorophyll‐*a*, DOC/POC, turbidity and hydrographic conditions. Consequently, direct quantitative evaluation of relationships between taxonomic variation and specific environmental variables was not possible (Martiny et al. 2015; Logares et al. 2020). Therefore, the present findings should be interpreted primarily as a descriptive assessment of regional community structuring rather than as evidence for specific environmental drivers or ecological mechanisms.

At each location and sampling period, three surface‐water samples were collected separately using individual sampling bottles and were treated as field replicates. However, each geographic region was represented by a single sampling location. Therefore, although the field replicates allowed within‐location variability to be assessed, the observed differences should primarily be interpreted as differences among the four sampled locations rather than as comprehensive estimates of variation across the broader coastal regions. In addition, this study included only two sampling periods (March and September), which represent two seasonal snapshots rather than a comprehensive characterization of annual seasonal variability. All samples were collected from the same surface layer using standardized sampling protocols and identical analytical procedures, thereby facilitating consistent comparisons among the sampled locations and periods. Nevertheless, the sampling dates differed slightly among locations because it was not logistically feasible to collect samples from all geographically distant locations on the same day. Although all samples were collected within the same sampling windows using standardized procedures, these differences in sampling dates should be acknowledged as a potential source of variability. Additional sampling across multiple locations within each region and repeated sampling within individual seasons would be required to evaluate broader regional and seasonal variability.

Within these limitations, diversity analysis, PERMANOVA and SIMPER documented differences in bacterial community composition and PPB assemblages among the four sampled coastal locations and between the March and September sampling periods. Indicator species analysis did not identify any statistically significant sampling‐location‐associated taxa after multiple‐testing correction. The present dataset therefore provides preliminary, location‐specific baseline information that may serve as a reference for future studies incorporating concurrent environmental metadata, broader spatial replication, repeated seasonal sampling and long‐term microbial monitoring. Accordingly, the findings should not be interpreted as a comprehensive regional baseline for Korean coastal waters or as evidence for specific environmental drivers or ecological mechanisms.

## 5 Conclusion

This study comparatively analysed surface‐water bacterial communities and putative PPB assemblages at four Korean coastal locations using a standardized sampling strategy and unified analytical framework. The results demonstrated differences in bacterial community composition among the four sampled locations and between the March and September sampling periods.

Across multiple analytical approaches, including diversity, taxonomic composition, PERMANOVA and SIMPER analyses, location‐associated variation was observed alongside differences between the March and September sampling periods. Spatial differentiation remained evident across both sampling periods, indicating that location‐associated differences in community composition were maintained under the conditions examined in this study. Location‐associated differences were primarily associated with variation at the class and genus levels within a higher‐level taxonomic framework dominated by Pseudomonadota. Within the PPB assemblage, *Vibrio* represented a common dominant taxon, whereas differences in the relative abundances of co‐occurring taxa contributed to distinct regional compositional patterns. Because concurrent environmental measurements were not available, the environmental drivers and ecological mechanisms underlying these patterns could not be directly evaluated. Furthermore, PPB classification was based on taxonomic affiliation and literature‐supported ecological associations and should not be interpreted as direct evidence of pathogenic strains, virulence potential or disease risk.

Overall, this study provides preliminary location‐specific baseline data describing bacterial community composition and PPB assemblages at four Korean coastal locations. These baseline data may serve as a reference for future microbial monitoring and studies incorporating broader spatial replication, repeated seasonal sampling and concurrent environmental measurements.

## Author Contributions

**Yu Jin Kim:** conceptualization, methodology, investigation, data curation, formal analysis, writing – original draft. **Min‐Jeong Kim:** investigation, data curation. **Seon Min Kim:** formal analysis, writing – review and editing. **Hyun Ju Ha:** formal analysis, validation. **Joon Sang Park:** formal analysis, writing – review and editing. **I. K. A. Agus Rini:** validation, formal analysis. **Sukchan Lee:** conceptualization, supervision, writing – review and editing. **Taek‐Kyun Lee:** writing – review and editing, supervision, conceptualization.

## Funding

This work was supported by the Korea Institute of Marine Science and Technology promotion (RS‐2021‐KS211475).

## Conflicts of Interest

The authors declare no conflicts of interest.

## Supporting information


**Table S1:** Geographic characteristics and sampling information of the study regions.
**Table S2:** Raw and retained sequencing read counts for each sample.
**Table S3:** Bacterial genera included in the putative pathogen‐associated bacterial taxa (PPB) dataset. Genera were selected based on published reports of pathogen‐associated or opportunistic species within each genus. Inclusion does not indicate that all members of the listed genera are pathogenic.
**Table S4:** Descriptive statistics and two‐way ANOVA results for alpha‐diversity indices of the total bacterial community and putative pathogen‐associated bacterial taxa (PPB) assemblage.
**Table S5:** PERMANOVA, simple‐effect and pairwise comparisons and tests of multivariate dispersion for total bacterial and putative pathogen‐associated bacterial taxa (PPB) assemblages. Bray–Curtis dissimilarities were analysed separately for the total bacterial community and putative pathogen‐associated bacterial taxa (PPB) assemblage. Full PERMANOVA models included Region, Season and their interaction. Follow‐up simple‐effect and pairwise analyses were conducted because the Region × Season interaction was significant. *p*‐Values from follow‐up analyses were adjusted using the Benjamini–Hochberg procedure.
**Table S6:** List of dominant bacterial taxa included in the taxonomic profiles. Taxonomic identification and mean relative abundances of the major phyla, classes and genera presented in Figure 4. For (D–F), PPB relative abundances are expressed as percentages of the total bacterial community rather than being renormalized within the PPB subset.
**Table S7:** Major taxa contributing to dissimilarity among PPB assemblages at the four sampled locations. Summary of SIMPER results showing the contributions of individual genera and their mean relative abundances at each location. Mean relative abundances are expressed as percentages of the total bacterial community and correspond to the abundance matrix used for SIMPER analysis; PPB abundances were not renormalized to 100% within the PPB subset. Only taxa cumulatively accounting for approximately 70% of the total dissimilarity are presented.
**Table S8:** Species‐level composition of selected putative pathogen‐associated bacterial taxa (PPB) detected in Korean coastal waters. Taxonomic assignments and mean relative abundances (%) of the putative species‐level PPB taxa presented in Figure 6 by sampling location and sampling period.
**Table S9:** Indicator taxa showing nominal associations with individual sampling locations or combinations of sampling locations. IndVal denotes the generalized indicator value (IndVal.g). Significance was evaluated using 9999 permutations and *p*‐values were adjusted across 181 taxa using the Benjamini–Hochberg procedure. None remained significant after adjustment (BH‐adjusted *p* ≥ 0.181).

## Data Availability

The raw 16S rRNA gene sequencing data have been deposited in the NCBI Sequence Read Archive (SRA) under accession number PRJNA1423342.
