# Supplementary material for: Spatial and Seasonal Structuring of Bacterial Communities and Putative Pathogen‐Associated Bacterial Taxa at Four Korean Coastal Locations
Source: Environ Microbiol Rep. 2026 Nov 3;18(6):e70431. doi: 10.1111/1758-2229.70431 (PMC13731945; doi:10.1111/1758-2229.70431)
Supplement: Supplementary file 1 [file EMI4-18-e70431-s001.docx]

**Spatial and Seasonal Structuring of Bacterial Communities and Putative Pathogen-Associated Bacterial Taxa at Four Korean Coastal Locations**

Yu Jin Kim^1,2^, Min-Jeong Kim^1^, Seon Min Kim^1,2^, Hyun Ju Ha^2,3^, Joon Sang Park^2,3^, IKA Agus Rini^4^, Sukchan Lee^4^, Taek-Kyun Lee^1,2,*^

^1^ Ecological Risk Research Department, Korea Institute of Ocean Science & Technology, Geoje 53201, Republic of Korea: rladbwls06069@kiost.ac.kr; min-jeong214@kiost.ac.kr; seonmin@kiost.ac.kr; tklee@kiost.ac.kr

^2^ Department of Ocean Science, University of Science and Technology, Daejeon 34113, Republic of Korea; rladbwls06069@kiost.ac.kr; seonmin@kiost.ac.kr; calltolove@kiost.ac.kr

^3^ Library of Marine Samples, Korea Institute of Ocean Science and Technology, Geoje 53201, Republic of Korea; jspark1101@kiost.ac.kr; calltolove@kiost.ac.kr

^4^ Department of Integrative Biotechnology, Sungkyunkwan University, Suwon 16419, Republic of Korea, cell4u@skku.edu

***** Correspondence: tklee@kiost.ac.kr

**Supplementary Information**

**Supplementary Tables**

**Table S1. Geographic characteristics and sampling information of the study regions.**

| **Region** | **Site**  **abbreviation** | **Geographic characteristics** | **Latitude (°N)** | **Longitude (°E)** | **Date** |
| --- | --- | --- | --- | --- | --- |
| Jejudo | JJ | Offshore island coast | 33.2278 | 126.5673 | Mar. 03 / Sep. 05, 2015 |
| Wando | WD | Semi-enclosed coastal waters | 34.3160 | 126.7560 | Mar. 08 / Sep. 10, 2015 |
| Somaemuldo | SM | Offshore island | 34.6193 | 128.5473 | Mar. 10 / Sep. 15, 2015 |
| Ulleungdo | UL | Oceanic island | 37.5330 | 130.8170 | Mar. 25 / Sep. 20, 2015 |

**Table S2. Raw and retained sequencing read counts for each sample.**

| **Sampling date** | **Region (Replicate)** | **Sample ID** | **Raw reads** | **Retained reads** |
| --- | --- | --- | --- | --- |
| 2015-03-08 | Wando-1 | B97 | 143,012 | 142,002 |
| 2015-03-08 | Wando-2 | B98 | 51,132 | 50,736 |
| 2015-03-08 | Wando-3 | B99 | 108,752 | 108,078 |
| 2015-03-03 | Jejudo-1 | B100 | 136,100 | 135,358 |
| 2015-03-03 | Jejudo-2 | B101 | 210,896 | 207,794 |
| 2015-03-03 | Jejudo-3 | B102 | 193,284 | 191,948 |
| 2015-03-10 | Somaemuldo-1 | B103 | 228,792 | 227,304 |
| 2015-03-10 | Somaemuldo-2 | B104 | 166,934 | 165,562 |
| 2015-03-10 | Somaemuldo-3 | B105 | 226,658 | 225,096 |
| 2015-03-25 | Ulleungdo-1 | B106 | 165,796 | 165,168 |
| 2015-03-25 | Ulleungdo-2 | B107 | 232,692 | 232,022 |
| 2015-03-25 | Ulleungdo-3 | B108 | 191,244 | 190,944 |
| 2015-09-10 | Wando-1 | B126 | 188,062 | 187,682 |
| 2015-09-10 | Wando-2 | B127 | 158,524 | 158,144 |
| 2015-09-10 | Wando-3 | B128 | 112,748 | 112,316 |
| 2015-09-15 | Somaemuldo-1 | B129 | 105,634 | 105,198 |
| 2015-09-15 | Somaemuldo-2 | B130 | 112,488 | 112,036 |
| 2015-09-15 | Somaemuldo-3 | B131 | 107,510 | 107,094 |
| 2015-09-20 | Ulleungdo-1 | B136 | 77,804 | 77,296 |
| 2015-09-20 | Ulleungdo-2 | B137 | 74,862 | 74,432 |
| 2015-09-20 | Ulleungdo-3 | B138 | 113,094 | 112,366 |
| 2015-09-05 | Jejudo-1 | B148 | 113,710 | 112,994 |
| 2015-09-05 | Jejudo-2 | B149 | 24,334 | 24,098 |
| 2015-09-05 | Jejudo-3 | B150 | 24,334 | 24,086 |

**Table S3. Bacterial genera included in the putative pathogen-associated bacterial taxa (PPB) dataset.** Genera were selected based on published reports of pathogen-associated or opportunistic species within each genus. Inclusion does not indicate that all members of the listed genera are pathogenic.

| **Genus** | **Evidence category** | **Basis for inclusion** | **Representative references** |
| --- | --- | --- | --- |
| *Vibrio* | Aquatic/marine pathogen-associated | Includes human and aquatic-animal pathogens. | Thompson et al., 2004; Baker-Austin et al., 2013 |
| *Shewanella* | Environmental opportunistic pathogen-associated | Includes aquatic species associated with opportunistic human infections. | Janda and Abbott, 2014; Cerbino et al., 2023 |
| *Tenacibaculum* | Aquatic/marine pathogen-associated | Includes pathogens causing tenacibaculosis in marine fish. | Avendaño-Herrera et al., 2006; Pérez-Pascual et al., 2017 |
| *Rhodococcus* | Environmental opportunistic pathogen-associated | Includes opportunistic animal and human pathogens. | Bell et al., 1998; Siavashifar et al., 2021 |
| *Bacteroides* | Human/animal pathogen-associated | Includes gut-associated opportunists causing extraintestinal infections. | Wexler, 2007; Murphy et al., 2011 |
| *Clostridium* | Human/animal pathogen-associated | Includes toxin-producing and opportunistic pathogens. | Wells and Wilkins, 1996 |
| *Pseudomonas* | Environmental opportunistic pathogen-associated | Includes opportunistic human and aquatic-animal pathogens. | Silby et al., 2011; Peix et al., 2018 |
| *Arcobacter* | Environmental opportunistic pathogen-associated | Includes water-associated taxa linked to human and animal disease. | Pérez-Cataluña et al., 2018; Ferreira et al., 2019 |
| *Parabacteroides* | Human-associated taxon with reported clinical associations | Includes gut-associated taxa occasionally causing extraintestinal infections. | Ezeji et al., 2021 |
| *Alistipes* | Human-associated taxon with reported clinical associations | Includes gut-associated taxa occasionally recovered from clinical infections. | Parker et al., 2020 |
| *Aeromonas* | Aquatic/marine pathogen-associated | Includes fish pathogens and opportunistic human pathogens. | Janda and Abbott, 2010; Fernández-Bravo and Figueras, 2020 |
| *Acinetobacter* | Environmental opportunistic pathogen-associated | Includes environmentally distributed clinical opportunists. | Doughari et al., 2011; Antunes et al., 2014 |
| *Klebsiella* | Human/animal pathogen-associated | Includes enteric and environmental opportunistic pathogens. | Podschun and Ullmann, 1998 |
| *Photobacterium* | Aquatic/marine pathogen-associated | Includes pathogens of marine animals and occasionally humans. | Rivas et al., 2013; Labella et al., 2017 |
| *Streptococcus* | Human/animal pathogen-associated | Includes human and aquatic-animal pathogens. | Vendrell et al., 2006 |
| *Prevotella* | Human/animal pathogen-associated | Includes mucosa-associated taxa linked to opportunistic infections. | Könönen and Gursoy, 2022 |
| *Haemophilus* | Human/animal pathogen-associated | Includes host-associated respiratory pathogens and opportunists. | Nørskov-Lauritsen, 2014 |
| *Francisella* | Aquatic/marine pathogen-associated | Includes pathogens causing francisellosis in fish and disease in mammals. | Colquhoun and Duodu, 2011 |
| *Staphylococcus* | Human/animal pathogen-associated | Includes host-associated opportunistic human and animal pathogens. | Becker et al., 2014 |
| *Fusobacterium* | Human/animal pathogen-associated | Includes taxa implicated in oral, systemic, and animal infections. | Brennan and Garrett, 2019 |
| *Enterobacter* | Environmental opportunistic pathogen-associated | Includes environmentally distributed clinical opportunists. | Davin-Regli et al., 2019 |
| *Moraxella* | Human/animal pathogen-associated | Includes respiratory and ocular pathogens and opportunists. | Murphy and Parameswaran, 2009 |
| *Mycobacterium* | Aquatic/marine pathogen-associated | Includes aquatic species associated with fish and opportunistic human infections. | Hashish et al., 2018 |

**Table S4. Descriptive statistics and two-way ANOVA results for alpha-diversity indices of the total bacterial community and putative pathogen-associated bacterial taxa (PPB) assemblage.**

A. Descriptive statistics of alpha-diversity indices

| Community Type | Location | Sampling period | Observed OTUs | Shannon | Gini–Simpson |
| --- | --- | --- | --- | --- | --- |
| Total bacteria | JJ | March | 142 ± 5 | 3.69 ± 0.30 | 0.91 ± 0.05 |
|  |  | September | 110 ± 17 | 3.46 ± 0.18 | 0.91 ± 0.02 |
|  | WD | March | 101 ± 17 | 2.47 ± 0.73 | 0.68 ± 0.20 |
|  |  | September | 108 ± 6 | 3.19 ± 0.63 | 0.84 ± 0.10 |
|  | SM | March | 128 ± 6 | 3.20 ± 0.29 | 0.88 ± 0.03 |
|  |  | September | 114 ± 11 | 3.62 ± 0.52 | 0.92 ± 0.06 |
|  | UL | March | 107 ± 5 | 3.37 ± 0.27 | 0.88 ± 0.02 |
|  |  | September | 266 ± 10 | 4.99 ± 0.06 | 0.98 ± 0.00 |
| PPB assemblage | JJ | March | 24.7 ± 2.1 | 2.64 ± 0.19 | 0.89 ± 0.02 |
|  |  | September | 27.0 ± 3.5 | 2.14 ± 0.59 | 0.75 ± 0.15 |
|  | WD | March | 12.7 ± 5.9 | 2.32 ± 0.52 | 0.88 ± 0.06 |
|  |  | September | 23.3 ± 1.2 | 1.43 ± 0.63 | 0.52 ± 0.24 |
|  | SM | March | 17.3 ± 0.6 | 2.53 ± 0.11 | 0.90 ± 0.02 |
|  |  | September | 13.7 ± 1.2 | 2.25 ± 0.14 | 0.86 ± 0.04 |
|  | UL | March | 15.7 ± 4.9 | 2.38 ± 0.40 | 0.87 ± 0.06 |
|  |  | September | 48.3 ± 5.9 | 3.69 ± 0.10 | 0.97 ± 0.01 |

B. Two-way ANOVA results

| **Community Type** | **Alpha-diversity index** | **Effect** | **F** | **p-value** |
| --- | --- | --- | --- | --- |
| Total bacterial | Observed OTUs | Sampling location | 58.35 | <0.001 |
|  |  | Season | 40.68 | <0.001 |
|  |  | Sampling location × Season | 87.03 | <0.001 |
|  | Shannon | Sampling location | 9.94 | <0.001 |
|  |  | Season | 12.76 | 0.003 |
|  |  | Sampling location × Season | 4.74 | 0.015 |
|  | Gini–Simpson | Sampling location | 6.46 | 0.005 |
|  |  | Season | 11.65 | 0.004 |
|  |  | Sampling location × Season | 2.86 | 0.070 |
| PPB assemblage | Observed OTUs | Sampling location | 24.24 | <0.001 |
|  |  | Season | 47.11 | <0.001 |
|  |  | Sampling location × Season | 27.02 | <0.001 |
|  | Shannon | Sampling location | 8.57 | 0.001 |
|  |  | Season | 0.29 | 0.596 |
|  |  | Sampling location × Season | 8.90 | 0.001 |
|  | Gini–Simpson | Sampling location | 7.62 | 0.002 |
|  |  | Season | 2.77 | 0.115 |
|  |  | Sampling location × Season | 9.03 | <0.001 |

**Table S5. PERMANOVA, simple-effect and pairwise comparisons, and tests of multivariate dispersion for total bacterial and putative pathogen-associated bacterial taxa (PPB) assemblages.** Bray–Curtis dissimilarities were analyzed separately for the total bacterial community and putative pathogen-associated bacterial taxa (PPB) assemblage. Full PERMANOVA models included Region, Season, and their interaction. Follow-up simple-effect and pairwise analyses were conducted because the Region × Season interaction was significant. P-values from follow-up analyses were adjusted using the Benjamini–Hochberg procedure.

A. PERMANOVA results

| **Community Type** | **Term** | **df** | **Sum of squares** | **R^2^** | **pseudo-F** | **p-value** |
| --- | --- | --- | --- | --- | --- | --- |
| Total bacteria | Region | 3 | 2.55 | 0.357 | 13.12 | <0.001 |
|  | Season | 1 | 1.49 | 0.209 | 23.06 | <0.001 |
|  | Region × Season | 3 | 2.06 | 0.289 | 10.61 | <0.001 |
|  | Residual | 16 | 1.04 | 0.145 | – | – |
|  | Total | 23 | 7.15 | 1.000 | – | – |
| PPB assemblage | Region | 3 | 1.38 | 0.273 | 7.37 | <0.001 |
|  | Season | 1 | 1.16 | 0.231 | 18.7 | <0.001 |
|  | Region × Season | 3 | 1.5 | 0.298 | 8.02 | <0.001 |
|  | Residual | 16 | 1 | 0.198 | – | – |
|  | Total | 23 | 5.03 | 1.000 | – | – |

B. Simple-effect and pairwise PERMANOVA results

(1) Location effects within each sampling period

| **Community Type** | **Sampling period** | **Sum of squares** | **R^2^** | **pseudo-F** | **BH-adjusted p** |
| --- | --- | --- | --- | --- | --- |
| Total bacteria | March | 2.2 | 0.776 | 9.25 | <0.001 |
|  | September | 2.42 | 0.857 | 15.96 | <0.001 |
| PPB assemblage | March | 0.57 | 0.500 | 2.66 | <0.001 |
|  | September | 2.3 | 0.844 | 14.47 | <0.001 |

(2) Pairwise comparisons among locations within each sampling period

| **Community Type** | **Sampling period** | **Comparison** | **R^2^** | **pseudo-F** | **BH-adjusted p** |
| --- | --- | --- | --- | --- | --- |
| Total bacteria | March | JJ vs WD | 0.757 | 12.47 | 0.1 |
|  |  | JJ vs SM | 0.670 | 8.13 | 0.1 |
|  |  | JJ vs UL | 0.430 | 3.02 | 0.1 |
|  |  | WD vs SM | 0.894 | 33.66 | 0.1 |
|  |  | WD vs UL | 0.689 | 8.87 | 0.1 |
|  |  | SM vs UL | 0.640 | 7.11 | 0.1 |
|  | September | JJ vs WD | 0.569 | 5.27 | 0.1 |
|  |  | JJ vs SM | 0.827 | 19.16 | 0.1 |
|  |  | JJ vs UL | 0.817 | 17.83 | 0.1 |
|  |  | WD vs SM | 0.789 | 15 | 0.1 |
|  |  | WD vs UL | 0.814 | 17.53 | 0.1 |
|  |  | SM vs UL | 0.866 | 25.80 | 0.1 |
| PPB assemblage | March | JJ vs WD | 0.311 | 1.81 | 0.1 |
|  |  | JJ vs SM | 0.46 | 3.41 | 0.1 |
|  |  | JJ vs UL | 0.377 | 2.42 | 0.1 |
|  |  | WD vs SM | 0.480 | 3.69 | 0.1 |
|  |  | WD vs UL | 0.318 | 1.87 | 0.1 |
|  |  | SM vs UL | 0.493 | 3.89 | 0.1 |
|  | September | JJ vs WD | 0.530 | 4.50 | 0.1 |
|  |  | JJ vs SM | 0.701 | 9.39 | 0.1 |
|  |  | JJ vs UL | 0.848 | 22.28 | 0.1 |
|  |  | WD vs SM | 0.719 | 10.23 | 0.1 |
|  |  | WD vs UL | 0.798 | 15.83 | 0.1 |
|  |  | SM vs UL | 0.801 | 16.11 | 0.1 |

(3) Sampling-period effects within each location

| **Community Type** | **Location** | **Sum of squares** | **R^2^** | **pseudo-F** | **BH-adjusted p** |
| --- | --- | --- | --- | --- | --- |
| Total bacteria | JJ | 0.65 | 0.662 | 7.83 | 0.1 |
|  | WD | 1.06 | 0.812 | 17.25 | 0.1 |
|  | SM | 0.93 | 0.940 | 63.04 | 0.1 |
|  | UL | 0.92 | 0.696 | 9.15 | 0.1 |
| PPB assemblage | JJ | 0.4 | 0.739 | 11.3 | 0.1 |
|  | WD | 0.59 | 0.648 | 7.37 | 0.1 |
|  | SM | 0.5 | 0.772 | 13.58 | 0.1 |
|  | UL | 1.17 | 0.752 | 12.13 | 0.1 |

C. Tests of homogeneity of multivariate dispersion

| **Community Type** | **Term** | **Sum of squares** | **pseudo-F** | **p-value** | **BH-adjusted p** |
| --- | --- | --- | --- | --- | --- |
| Total bacteria | Region | 0.02 | 0.87 | 0.47 | 0.931 |
|  | Season | 0.00 | 0.01 | 0.93 | 0.931 |
|  | Region × Season | 0.1 | 0.73 | 0.65 | 0.931 |
| PPB assemblage | Region | 0.18 | 6.45 | 0.004 | 0.012 |
|  | Season | 0.1 | 3.03 | 0.09 | 0.128 |
|  | Region × Season | 0.1 | 1.92 | 0.13 | 0.128 |

**Table S6. List of dominant bacterial taxa included in the taxonomic profiles.** Taxonomic identification and mean relative abundances of the major phyla, classes, and genera presented in Figure 4. For panels D–F, PPB relative abundances are expressed as percentages of the total bacterial community rather than being renormalized within the PPB subset.

A. Total bacterial phyla.

| **Season** | **March** | | | | **September** | | | |
| --- | --- | --- | --- | --- | --- | --- | --- | --- |
| Region | JJ | WD | SM | UL | JJ | WD | SM | UL |
| Pseudomonadota | 96.95 | 96.07 | 93.98 | 90.95 | 94.36 | 95.99 | 87.56 | 63.89 |
| Bacteroidota | 1.87 | 3.15 | 5.04 | 7.04 | 5.34 | 3.45 | 11.81 | 12.39 |
| Actinomycetota | 0.31 | 0.35 | 0.11 | 0.27 | 0.12 | 0.28 | 0.35 | 21.32 |
| Others(<1%) | 0.86 | 0.42 | 0.86 | 1.75 | 0.18 | 0.27 | 0.28 | 2.39 |

B. Total bacterial classes.

| **Season** | **March** | | | | **September** | | | |
| --- | --- | --- | --- | --- | --- | --- | --- | --- |
| Region | JJ | WD | SM | UL | JJ | WD | SM | UL |
| Gammaproteobacteria | 75.04 | 78.65 | 88.96 | 55.71 | 51.16 | 70.9 | 62.6 | 40.72 |
| Alphaproteobacteria | 21.81 | 17.37 | 5.01 | 35 | 43.03 | 25.05 | 24.88 | 19.67 |
| Bacteroidia | 1.21 | 1.32 | 0.97 | 5.74 | 5.26 | 0.72 | 5.11 | 10.12 |
| Actinomycetes | 0.23 | 0.3 | 0.09 | 0.06 | 0.11 | 0.18 | 0.15 | 20.94 |
| Flavobacteriia | 0.52 | 1.3 | 4.04 | 1.24 | 0.09 | 0.22 | 5.74 | 2.28 |
| Others(<1%) | 1.18 | 1.05 | 0.93 | 2.25 | 0.36 | 2.92 | 1.52 | 6.27 |

C. Total bacterial genera.

| **Season** | **March** | | | | **September** | | | |
| --- | --- | --- | --- | --- | --- | --- | --- | --- |
| Region | JJ | WD | SM | UL | JJ | WD | SM | UL |
| *Pseudoalteromonas* | 50.75 | 0.24 | 48.98 | 15.13 | 9.4 | 15.02 | 55.86 | 5.54 |
| *Vibrio* | 16.96 | 1.67 | 2.67 | 6.82 | 39.16 | 53.69 | 4.01 | 1.49 |
| *Psychromonas* | 0.42 | 72.19 | 30.61 | 15.32 | 0.01 | 0.46 | 0 | 0.17 |
| *Pelagibacter* | 11.45 | 5.15 | 1.51 | 26.39 | 20.22 | 5.78 | 4.56 | 4.69 |
| *Sulfitobacter* | 0.59 | 0.68 | 0.84 | 2.99 | 1.1 | 1.07 | 8.61 | 2.66 |
| *Leucobacter* | 0 | 0 | 0 | 0 | 0.02 | 0 | 0 | 16.32 |
| *Planktomarina* | 2.33 | 7.03 | 1.27 | 1.06 | 0.01 | 2.87 | 0.11 | 0.15 |
| *Leisingera* | 1.6 | 0.16 | 0 | 0.02 | 6.8 | 3.83 | 0.03 | 0.94 |
| *Polaribacter* | 0.13 | 0.41 | 0.13 | 3.51 | 5.01 | 0.06 | 1.85 | 1.54 |
| *Colwellia* | 0.68 | 0.78 | 3.75 | 5.09 | 0 | 0.02 | 0 | 0.04 |
| Others(<1%) | 15.09 | 11.7 | 10.22 | 23.67 | 18.26 | 17.19 | 24.98 | 66.45 |

D. Putative pathogen-associated taxa phyla.

| **Season** | **March** | | | | **September** | | | |
| --- | --- | --- | --- | --- | --- | --- | --- | --- |
| Region | JJ | WD | SM | UL | JJ | WD | SM | UL |
| Pseudomonadota | 17.37 | 1.88 | 2.84 | 9.56 | 39.43 | 53.84 | 4.18 | 7.56 |
| Bacteroidota | 0.45 | 0.41 | 0.17 | 0.05 | 0.01 | 0.08 | 0.03 | 0.53 |
| Actinomycetota | 0 | 0.15 | 0.02 | 0 | 0.06 | 0.21 | 0.31 | 0.21 |
| Others(<0.1%) | 0.05 | 0.03 | 0.06 | 0.07 | 0.01 | 0.01 | 0.03 | 0.54 |

E. Putative pathogen-associated taxa classes.

| **Season** | **March** | | | | **September** | | | |
| --- | --- | --- | --- | --- | --- | --- | --- | --- |
| Region | JJ | WD | SM | UL | JJ | WD | SM | UL |
| Gammaproteobacteria | 17.3 | 1.88 | 2.84 | 9.42 | 39.34 | 53.83 | 4.16 | 6.48 |
| Bacteroidia | 0.34 | 0.41 | 0.17 | 0.05 | 0.01 | 0.03 | 0.03 | 0.42 |
| Campylobacteria | 0.01 | 0 | 0 | 0 | 0.11 | 0 | 0 | 0.84 |
| Others(<0.1%) | 0.22 | 0.19 | 0.08 | 0.21 | 0.06 | 0.28 | 0.37 | 1.1 |

F. Putative pathogen-associated taxa genera.

| **Season** | **March** | | | | **September** | | | |
| --- | --- | --- | --- | --- | --- | --- | --- | --- |
| Region | JJ | WD | SM | UL | JJ | WD | SM | UL |
| *Vibrio* | 16.96 | 1.67 | 2.67 | 6.82 | 39.16 | 53.69 | 4.01 | 1.49 |
| *Pseudomonas* | 0.07 | 0.2 | 0.02 | 2.63 | 0 | 0.06 | 0.07 | 0.4 |
| *Acinetobacter* | 0 | 0 | 0 | 0 | 0.04 | 0 | 0.04 | 2.75 |
| *Arcobacter* | 0.01 | 0 | 0 | 0 | 0.11 | 0 | 0 | 0.84 |
| *Shewanella* | 0.29 | 0 | 0.14 | 0 | 0.01 | 0.06 | 0 | 0.39 |
| *Rhodococcus* | 0 | 0.15 | 0.02 | 0 | 0.06 | 0.21 | 0.31 | 0.09 |
| *Aeromonas* | 0.01 | 0 | 0 | 0 | 0.05 | 0.01 | 0 | 0.77 |
| *Tenacibaculum* | 0.29 | 0.26 | 0 | 0.05 | 0 | 0.07 | 0.01 | 0.13 |
| Others(<0.1%) | 0.23 | 0.21 | 0.24 | 0.18 | 0.09 | 0.04 | 0.1 | 1.98 |

**Table S7. Major taxa contributing to dissimilarity among PPB assemblages at the four sampled locations.** Summary of SIMPER results showing the contributions of individual genera and their mean relative abundances at each location. Mean relative abundances are expressed as percentages of the total bacterial community and correspond to the abundance matrix used for SIMPER analysis; PPB abundances were not renormalized to 100% within the PPB subset. Only taxa cumulatively accounting for approximately 70% of the total dissimilarity are presented.

| Comparison | Taxon | Contribution | Cumulative | Mean  relative abundance (A) | Mean  relative abundance (B) |
| --- | --- | --- | --- | --- | --- |
| JJ vs SM | *Vibrio* | 0.65 | 0.65 | 28.06 | 3.34 |
|  | *Shewanella* | 0.01 | 0.66 | 0.15 | 0.07 |
|  | *Tenacibaculum* | 0.01 | 0.67 | 0.15 | 0.01 |
|  | *Rhodococcus* | 0.01 | 0.68 | 0.03 | 0.17 |
|  | *Bacteroides* | 0 | 0.68 | 0.07 | 0.02 |
|  | *Clostridium* | 0 | 0.68 | 0.01 | 0.04 |
|  | *Pseudomonas* | 0 | 0.68 | 0.04 | 0.04 |
|  | *Arcobacter* | 0 | 0.68 | 0.06 | 0 |
|  | *Parabacteroides* | 0 | 0.69 | 0 | 0.03 |
|  | *Alistipes* | 0 | 0.69 | 0.01 | 0.02 |
|  | *Aeromonas* | 0 | 0.69 | 0.03 | 0 |
|  | *Acinetobacter* | 0 | 0.69 | 0.02 | 0.02 |
|  | *Klebsiella* | 0 | 0.69 | 0 | 0.02 |
|  | *Photobacterium* | 0 | 0.69 | 0.04 | 0 |
|  | *Streptococcus* | 0 | 0.69 | 0.02 | 0 |
|  | *Prevotella* | 0 | 0.69 | 0 | 0.02 |
|  | *Haemophilus* | 0 | 0.7 | 0.01 | 0 |
|  | *Francisella* | 0 | 0.7 | 0 | 0.01 |
|  | *Staphylococcus* | 0 | 0.7 | 0 | 0 |
|  | *Fusobacterium* | 0 | 0.7 | 0 | 0 |
| JJ vs UL | *Vibrio* | 0.54 | 0.54 | 28.06 | 4.16 |
|  | *Acinetobacter* | 0.05 | 0.59 | 0.02 | 1.37 |
|  | *Pseudomonas* | 0.05 | 0.63 | 0.04 | 1.51 |
|  | *Arcobacter* | 0.01 | 0.65 | 0.06 | 0.42 |
|  | *Aeromonas* | 0.01 | 0.66 | 0.03 | 0.38 |
|  | *Clostridium* | 0.01 | 0.67 | 0.01 | 0.24 |
|  | *Shewanella* | 0.01 | 0.68 | 0.15 | 0.2 |
|  | *Tenacibaculum* | 0.01 | 0.68 | 0.15 | 0.09 |
|  | *Enterobacter* | 0.01 | 0.69 | 0 | 0.16 |
|  | *Photobacterium* | 0.01 | 0.7 | 0.04 | 0.17 |
|  | *Bacteroides* | 0.01 | 0.7 | 0.07 | 0.15 |
|  | *Moraxella* | 0 | 0.71 | 0 | 0.12 |
|  | *Klebsiella* | 0 | 0.71 | 0 | 0.07 |
|  | *Mycobacterium* | 0 | 0.71 | 0 | 0.06 |
|  | *Rhodococcus* | 0 | 0.71 | 0.03 | 0.05 |
|  | *Prevotella* | 0 | 0.71 | 0 | 0.05 |
|  | *Streptococcus* | 0 | 0.72 | 0.02 | 0.04 |
|  | *Francisella* | 0 | 0.72 | 0 | 0.02 |
|  | *Alistipes* | 0 | 0.72 | 0.01 | 0 |
|  | *Haemophilus* | 0 | 0.72 | 0.01 | 0 |
|  | *Parabacteroides* | 0 | 0.72 | 0 | 0 |
| JJ vs WD | *Vibrio* | 0.59 | 0.59 | 28.06 | 27.68 |
|  | *Tenacibaculum* | 0.01 | 0.6 | 0.15 | 0.17 |
|  | *Shewanella* | 0.01 | 0.6 | 0.15 | 0.03 |
|  | *Rhodococcus* | 0 | 0.61 | 0.03 | 0.18 |
|  | *Pseudomonas* | 0 | 0.61 | 0.04 | 0.13 |
|  | *Prevotella* | 0 | 0.61 | 0 | 0.07 |
|  | *Bacteroides* | 0 | 0.62 | 0.07 | 0.01 |
|  | *Arcobacter* | 0 | 0.62 | 0.06 | 0 |
|  | *Photobacterium* | 0 | 0.62 | 0.04 | 0.02 |
|  | *Aeromonas* | 0 | 0.62 | 0.03 | 0 |
|  | *Streptococcus* | 0 | 0.62 | 0.02 | 0.02 |
|  | *Clostridium* | 0 | 0.62 | 0.01 | 0.01 |
|  | *Alistipes* | 0 | 0.62 | 0.01 | 0 |
|  | *Acinetobacter* | 0 | 0.62 | 0.02 | 0 |
|  | *Parabacteroides* | 0 | 0.62 | 0 | 0 |
|  | *Haemophilus* | 0 | 0.62 | 0.01 | 0 |
| SM vs UL | *Vibrio* | 0.21 | 0.21 | 3.34 | 4.16 |
|  | *Acinetobacter* | 0.11 | 0.32 | 0.02 | 1.37 |
|  | *Pseudomonas* | 0.1 | 0.42 | 0.04 | 1.51 |
|  | *Arcobacter* | 0.03 | 0.46 | 0 | 0.42 |
|  | *Aeromonas* | 0.03 | 0.49 | 0 | 0.38 |
|  | *Clostridium* | 0.02 | 0.51 | 0.04 | 0.24 |
|  | *Shewanella* | 0.02 | 0.52 | 0.07 | 0.2 |
|  | *Enterobacter* | 0.01 | 0.53 | 0 | 0.16 |
|  | *Photobacterium* | 0.01 | 0.55 | 0 | 0.17 |
|  | *Rhodococcus* | 0.01 | 0.56 | 0.17 | 0.05 |
|  | *Bacteroides* | 0.01 | 0.57 | 0.02 | 0.15 |
|  | *Moraxella* | 0.01 | 0.58 | 0 | 0.12 |
|  | *Tenacibaculum* | 0.01 | 0.59 | 0.01 | 0.09 |
|  | *Klebsiella* | 0.01 | 0.59 | 0.02 | 0.07 |
|  | *Prevotella* | 0 | 0.6 | 0.02 | 0.05 |
|  | *Mycobacterium* | 0 | 0.6 | 0 | 0.06 |
|  | *Streptococcus* | 0 | 0.61 | 0 | 0.04 |
|  | *Parabacteroides* | 0 | 0.61 | 0.03 | 0 |
|  | *Francisella* | 0 | 0.61 | 0.01 | 0.02 |
|  | *Alistipes* | 0 | 0.61 | 0.02 | 0 |
|  | *Staphylococcus* | 0 | 0.61 | 0 | 0 |
|  | *Fusobacterium* | 0 | 0.61 | 0 | 0 |
| SM vs WD | *Vibrio* | 0.64 | 0.64 | 3.34 | 27.68 |
|  | *Tenacibaculum* | 0.02 | 0.66 | 0.01 | 0.17 |
|  | *Rhodococcus* | 0.02 | 0.67 | 0.17 | 0.18 |
|  | *Pseudomonas* | 0.01 | 0.69 | 0.04 | 0.13 |
|  | *Prevotella* | 0.01 | 0.7 | 0.02 | 0.07 |
|  | *Shewanella* | 0.01 | 0.7 | 0.07 | 0.03 |
|  | *Clostridium* | 0 | 0.71 | 0.04 | 0.01 |
|  | *Parabacteroides* | 0 | 0.71 | 0.03 | 0 |
|  | *Klebsiella* | 0 | 0.71 | 0.02 | 0 |
|  | *Acinetobacter* | 0 | 0.72 | 0.02 | 0 |
|  | *Bacteroides* | 0 | 0.72 | 0.02 | 0.01 |
|  | *Photobacterium* | 0 | 0.72 | 0 | 0.02 |
|  | *Alistipes* | 0 | 0.72 | 0.02 | 0 |
|  | *Streptococcus* | 0 | 0.73 | 0 | 0.02 |
|  | *Francisella* | 0 | 0.73 | 0.01 | 0 |
|  | *Staphylococcus* | 0 | 0.73 | 0 | 0 |
|  | *Fusobacterium* | 0 | 0.73 | 0 | 0 |
|  | *Aeromonas* | 0 | 0.73 | 0 | 0 |
| UL vs WD | *Vibrio* | 0.52 | 0.52 | 4.16 | 27.68 |
|  | *Acinetobacter* | 0.07 | 0.6 | 1.37 | 0 |
|  | *Pseudomonas* | 0.07 | 0.66 | 1.51 | 0.13 |
|  | *Arcobacter* | 0.02 | 0.69 | 0.42 | 0 |
|  | *Aeromonas* | 0.02 | 0.71 | 0.38 | 0 |
|  | *Clostridium* | 0.01 | 0.72 | 0.24 | 0.01 |
|  | *Tenacibaculum* | 0.01 | 0.73 | 0.09 | 0.17 |
|  | *Shewanella* | 0.01 | 0.74 | 0.2 | 0.03 |
|  | *Enterobacter* | 0.01 | 0.75 | 0.16 | 0 |
|  | *Photobacterium* | 0.01 | 0.76 | 0.17 | 0.02 |
|  | *Bacteroides* | 0.01 | 0.77 | 0.15 | 0.01 |
|  | *Rhodococcus* | 0.01 | 0.78 | 0.05 | 0.18 |
|  | *Moraxella* | 0.01 | 0.78 | 0.12 | 0 |
|  | *Prevotella* | 0.01 | 0.79 | 0.05 | 0.07 |
|  | *Klebsiella* | 0 | 0.79 | 0.07 | 0 |
|  | *Mycobacterium* | 0 | 0.8 | 0.06 | 0 |
|  | *Streptococcus* | 0 | 0.8 | 0.04 | 0.02 |
|  | *Francisella* | 0 | 0.8 | 0.02 | 0 |

**Table S8. Species-level composition of selected putative pathogen-associated bacterial taxa (PPB) detected in Korean coastal waters.** Taxonomic assignments and mean relative abundances (%) of the putative species-level PPB taxa presented in Figure 6 by sampling location and sampling period.

| **Season** | **March** | | | | **September** | | | |
| --- | --- | --- | --- | --- | --- | --- | --- | --- |
| Region | JJ | WD | SM | UL | JJ | WD | SM | UL |
| *Vibrio tasmaniensis* | 2.07 | 0.76 | 1.16 | 1.62 | 4.93 | 6.42 | 1.18 | 0.23 |
| *Vibrio pomeroyi* | 1.86 | 0.48 | 0.48 | 0.75 | 2.16 | 1.29 | 0.65 | 0.44 |
| *Vibrio chagasii* | 0.17 | 0.00 | 0.00 | 0.00 | 2.37 | 0.28 | 1.32 | 0.13 |
| *Vibrio hemicentroti* | 1.88 | 0.46 | 0.72 | 1.05 | 0.59 | 0.46 | 0.26 | 0.23 |
| *Vibrio ordalii* | 0.00 | 0.00 | 0.00 | 0.00 | 0.00 | 2.35 | 0.29 | 0.00 |
| *Vibrio lentus* | 1.34 | 0.53 | 0.69 | 0.78 | 0.76 | 1.18 | 0.28 | 0.00 |
| *Vibrio splendidus* | 1.58 | 0.61 | 0.32 | 0.62 | 0.35 | 0.15 | 0.31 | 0.00 |
| *Vibrio kanaloae* | 0.19 | 0.00 | 0.00 | 0.00 | 1.18 | 1.36 | 0.15 | 0.00 |
| *Pseudomonas guineae* | 0.11 | 0.43 | 0.07 | 1.25 | 0.00 | 0.00 | 0.00 | 0.00 |
| *Acinetobacter parvus* | 0.00 | 0.00 | 0.00 | 0.00 | 0.00 | 0.00 | 0.00 | 1.29 |
| *Vibrio atypicus* | 0.80 | 0.00 | 0.00 | 0.90 | 0.41 | 0.00 | 0.00 | 0.14 |
| *Vibrio cortegadensis* | 0.11 | 0.00 | 0.00 | 0.00 | 0.22 | 0.99 | 0.11 | 0.71 |
| *Vibrio toranzoniae* | 0.22 | 0.00 | 0.00 | 0.34 | 0.88 | 0.39 | 0.11 | 0.00 |
| *Arcobacter cryaerophilus* | 0.09 | 0.00 | 0.00 | 0.00 | 0.28 | 0.00 | 0.00 | 0.89 |

**Table S9. Indicator taxa showing nominal associations with individual sampling locations or combinations of sampling locations.** IndVal denotes the generalized indicator value (IndVal.g). Significance was evaluated using 9,999 permutations, and p-values were adjusted across 181 taxa using the Benjamini–Hochberg procedure. None remained significant after adjustment (BH-adjusted p ≥ 0.181).

| **Associated sampling location(s)** | **OTU** | **Putative taxonomic assignment** | **IndVal** | **Raw p-value** | **BH-adjusted**  **p-value** |
| --- | --- | --- | --- | --- | --- |
| JJ+UL | OTU057 | *Vibrio atypicus* | 0.913 | 0.0010 | 0.181 |
| JJ | OTU262 | *Vibrio comitans* | 0.831 | 0.0021 | 0.190 |
| UL | OTU136 | *Pseudomonas pelagia* | 0.857 | 0.0032 | 0.193 |
| JJ | OTU415 | *Vibrio tapetis* | 0.816 | 0.0062 | 0.281 |
| JJ+WD | OTU031 | *Vibrio kanaloae* | 0.814 | 0.0121 | 0.322 |
| JJ+WD | OTU144 | *Vibrio breoganii* | 0.795 | 0.0132 | 0.322 |
| UL | OTU126 | *Pseudomonas sabulinigri* | 0.702 | 0.0373 | 0.322 |
| JJ | OTU378 | *Vibrio pelagius* | 0.655 | 0.0403 | 0.322 |
| UL | OTU054 | *Acinetobacter parvus* | 0.707 | 0.0409 | 0.322 |
| UL | OTU113 | *Acinetobacter tjernbergiae* | 0.688 | 0.0409 | 0.322 |
| UL | OTU142 | *Aeromonas sharmana* | 0.707 | 0.0409 | 0.322 |
| UL | OTU164 | *Aeromonas punctata* | 0.707 | 0.0409 | 0.322 |
| UL | OTU189 | *Acinetobacter junii* | 0.707 | 0.0409 | 0.322 |
| UL | OTU191 | *Moraxella osloensis* | 0.707 | 0.0409 | 0.322 |
| UL | OTU227 | *Shewanella pacifica* | 0.681 | 0.0409 | 0.322 |
| UL | OTU233 | *Clostridium puniceum* | 0.707 | 0.0409 | 0.322 |
| UL | OTU255 | *Bacteroides graminisolvens* | 0.707 | 0.0409 | 0.322 |
| UL | OTU284 | *Bacteroides luti* | 0.707 | 0.0409 | 0.322 |
| UL | OTU315 | *Vibrio ezurae* | 0.672 | 0.0409 | 0.322 |
| WD | OTU019 | *Vibrio ordalii* | 0.702 | 0.0415 | 0.322 |
| SM | OTU405 | *Shewanella livingstonensis* | 0.707 | 0.0416 | 0.322 |
| SM | OTU447 | *Shewanella arctica* | 0.707 | 0.0416 | 0.322 |
| WD | OTU364 | *Tenacibaculum adriaticum* | 0.707 | 0.042 | 0.322 |
| JJ | OTU174 | *Shewanella kaireitica* | 0.69 | 0.0427 | 0.322 |
